# Genome-scale analyses of butanol tolerance in *Saccharomyces cerevisiae* reveal an essential role of protein degradation

**DOI:** 10.1186/1754-6834-6-48

**Published:** 2013-04-03

**Authors:** Daniel González-Ramos, Marcel van den Broek, Antonius JA van Maris, Jack T Pronk, Jean-Marc G Daran

**Affiliations:** 1 Department of Biotechnology, Delft University of Technology, Julianalaan 67, Delft 2628 BC, The Netherlands; 2 Kluyver Centre for Genomics of Industrial Fermentation, P.O. Box 5057, Delft 2600 GA, The Netherlands; 3 Platform for Green Synthetic Biology, P.O. Box 5057, Delft 2600 GA, The Netherlands

**Keywords:** *Saccharomyces cerevisiae*, Butanol tolerance, Evolutionary engineering, Deletion collection screening, Whole genome sequencing, Proteasome, Multivesicular bodies

## Abstract

**Background:**

*n*-Butanol and isobutanol produced from biomass-derived sugars are promising renewable transport fuels and solvents. *Saccharomyces cerevisiae* has been engineered for butanol production, but its high butanol sensitivity poses an upper limit to product titers that can be reached by further pathway engineering. A better understanding of the molecular basis of butanol stress and tolerance of *S. cerevisiae* is important for achieving improved tolerance.

**Results:**

By combining a screening of the haploid *S. cerevisiae* knock-out library, gene overexpression, and genome analysis of evolutionary engineered *n*-butanol-tolerant strains, we established that protein degradation plays an essential role in tolerance. Strains deleted in genes involved in the ubiquitin-proteasome system and in vacuolar degradation of damaged proteins showed hypersensitivity to *n-*butanol. Overexpression of YLR224W, encoding the subunit responsible for the recognition of damaged proteins of an ubiquitin ligase complex, resulted in a strain with a higher *n*-butanol tolerance. Two independently evolved *n*-butanol-tolerant strains carried different mutations in both *RPN4* and *RTG1*, which encode transcription factors involved in the expression of proteasome and peroxisomal genes, respectively. Introduction of these mutated alleles in the reference strain increased butanol tolerance, confirming their relevance in the higher tolerance phenotype. The evolved strains, in addition to *n*-butanol, were also more tolerant to 2-butanol, isobutanol and 1-propanol, indicating a common molecular basis for sensitivity and tolerance to C3 and C4 alcohols.

**Conclusions:**

This study shows that maintenance of protein integrity plays an essential role in butanol tolerance and demonstrates new promising targets to engineer *S. cerevisiae* for improved tolerance.

## Background

Four-carbon alcohols, including *n-*butanol, produced from renewable biomass, are promising alternatives to ethanol as biofuels: they are less volatile, less hygroscopic and less corrosive than ethanol, and have higher energy content. They can also be added to gasoline as a fuel additive or even replace it completely without modification of the existing car engines [1,2] and are compatible with the existing infrastructure for gasoline distribution [1].

*n*-Butanol can be naturally produced by some *Clostridium* species through the so called ABE fermentation, a process that yields acetone, butanol and ethanol [3]. ‘Biobutanol’ has been historically produced through this process [4], however, it exhibits several drawbacks that limit its current application for large scale production. *Clostridium* species are strictly anaerobic and slow growing microorganisms [5]. The low *n-*butanol yield and the production of by-products such as acetone and ethanol also affect butanol production negatively. Although attempts have been made to engineer *Clostridium* in order to overcome these limitations [3,5,6], its complex physiology and lack of efficient genetic tools make it difficult to engineer [5].

The limitations of clostridial *n*-butanol production were an incentive to develop and use alternative hosts. To this end, the genes encoding the *n*-butanol biosynthetic pathway from *C. acetobutilicum* have been expressed in different microorganisms. In engineered *Escherichia coli* strains, *n*-butanol titers of up to 600 mg/l were reached via this strategy [7-9]. *Pseudomonas putida*, *Bacillus subtilis* and *Lactobacillus brevis* have also been used as butanol-producing hosts, although the concentrations obtained were lower than those reached by *E. coli*[10,11]. Similarly, *S. cerevisiae* has been engineered for *n*-butanol production by substituting the clostridial enzymes by isozymes from different microorganisms, however, the concentration obtained remained very low (2.5 mg/l) [12]. Interestingly, *S. cerevisiae* can also produce *n*-butanol from an endogenous pathway that involves norvaline as an intermediate, which may offer new, interesting options for pathway engineering [13-15].

In addition to pathway engineering, product toxicity represents a major challenge in microbial butanol production [16,17]. As an organic solvent, *n*-butanol tends to partition into biological membranes, thereby increasing their fluidity and changing their structures [17-19]. Membrane functions are severely hampered in the presence of *n*-butanol; cells lose the ability to maintain internal pH due to the increased proton permeability of the cytoplasmic membrane and inhibition of the membrane ATPase [20,21]. The increase in membrane fluidity also results in a loss of intracellular molecules such as proteins, RNA and ATP [10] and glucose uptake is strongly impeded [20]. *In situ* product recovery systems allow the efficient removal of *n*-butanol and thereby reduce toxicity to the cells [5,22], however, they add substantial capital and operating costs to the process. These costs would be reduced with a higher operating concentration of *n*-butanol, and thus more tolerant strains would greatly improve process economics [3,16,17].

Improvement of *n*-butanol tolerance has been explored in both *E. coli* and *S. cerevisiae*. The overexpression in *E. coli* of genes related to iron transport and metabolism increase isobutanol tolerance as well as genes involved in membrane functions, amino acid transport, sugar transport and stress response, played a role in tolerance [23]. In another study, genes related to the synthesis of glucosamine-6-phosphate (precursor of peptidoglycan and lipopolysaccharide), multidrug efflux system, degradation of L-cysteine and L-tryptophan, and galactitol metabolism were identified as potential targets for further engineering of butanol tolerance in *E. coli*[24]. The overexpression in *E. coli* of GroEL/GroES, molecular chaperones that prevent protein aggregation under stress conditions and assist in protein folding, also resulted in strains with an improved butanol tolerance [25]. However, the levels of tolerance obtained so far in *E. coli* through different strategies do not match its production capacity and therefore tolerance needs to be further investigated and improved.

*S. cerevisiae* is known for its high tolerance to alcohols [17,26] and low pH [27], which is especially relevant when fermenting lignocellulosic material. Several groups attempted to increase *n*-butanol tolerance in *S. cerevisiae* by modifying genes involved in multidrug resistance [28], cell wall integrity [29], high osmolarity response [30], filamentous growth [31] and amino acid starvation [32]. Some of the modified strains showed increased biomass yield or growth rate relative to their reference in the presence of specific *n*-butanol concentrations. However, the maximum *n*-butanol concentration that allows growth in *S. cerevisiae* has not been increased and the mechanisms of butanol toxicity remain largely unknown.

**Figure 1:**
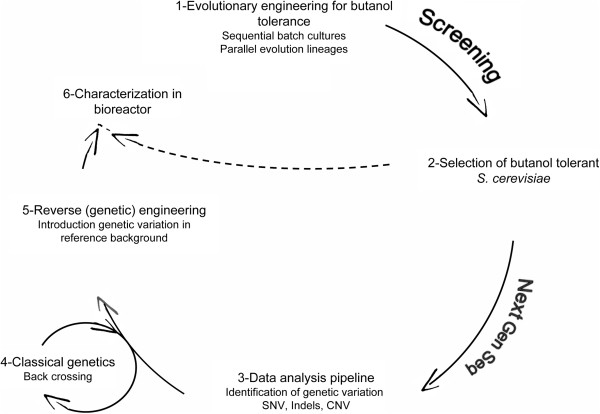
**Reverse engineering cycle for butanol tolerance in *S. cerevisiae*.** The figure represents the evolutionary engineering strategy used in this study. It comprises three phases a) generation of biodiversity by evolutionary engineering and screening for improved isolates (1- and 2-), b) analysis of evolved genomes and identification of genetic variations (SNV: Single Nucleotide Variation, INDELS: INsertion - DELetion and CNV: Copy Number Variation) (3-) and c) reverse genetic engineering of detected variation in a “naive” genetic background and characterization of the engineered strain (5-, 6-). Prioritization of the variations prior reintroduction in naive reference was guided by sequence analysis of offspring from three consecutive back crossing (4-).

The aim of the present study was to identify the metabolic functions associated with *n*-butanol tolerance in *S. cerevisiae.* Our strategy used a combination of two complementary genome-scale approaches: a screening of the haploid non-essential gene knockout collection [33,34] and a laboratory evolution approach followed by whole genome resequencing (Figure 1) [35]. In the experimental design, special attention was paid to avoid evaporation of *n*-butanol, which otherwise can obscure the results of screening studies. The target genes identified in both approaches were characterized and eventually enabled us to functionally map new mechanisms involved in *n*-butanol tolerance in *S. cerevisiae*

## Results

### Experimental design and evaluation of *n*-butanol tolerance of *S. cerevisiae* strains BY4741 and CEN.PK113-7D

**Figure 2:**
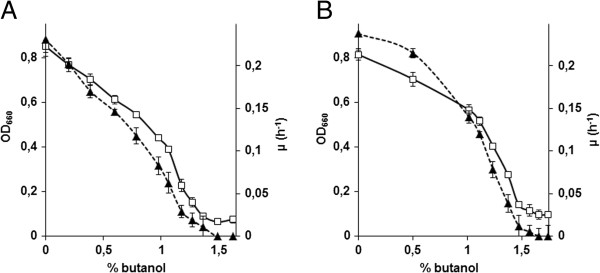
**Specific growth rate and final OD**_**660 **_**measured after 48 h of incubation of the reference strains BY4741 (A) and CEN.PK113-7D (B) in 96 well plates in synthetic medium containing *n*-butanol concentrations ranging from 0 to 1.9% (v/v).** □: OD_660,_ ▲: μ (h^-1^). The data presented are average and standard deviation of at least eight biological replicates.

To assess the impact of *n*-butanol on growth rate and biomass yield, *S. cerevisiae* strains BY4741 and CEN.PK113-7D were grown in 96-well plates containing synthetic medium with *n*-butanol concentrations ranging from 0% to 1.9% (v/v). In unsealed plates, *n*-butanol concentrations changed significantly and, from start to end of the culture, decreased by 50% irrespective of the initial concentration. To minimize butanol evaporation, plates were sealed with a gas-impermeable film, thereby reducing evaporation to a maximum of 10% after 48 h. Application of the gas-impermeable film also prevented oxygen transfer and, thereby oxidation of butanol to butyric acid. Plates were incubated for 48 h at 30°C and OD_660_ was recorded every 30 min. The inhibitory effect of *n*-butanol was moderate up to a concentration of 1%. At that concentration, the OD_660_ values of strains BY4741 and CEN.PK113-7D in stationary phase were 50% and 30% lower, respectively, than under non-stressed conditions (Figure 2A and B). Above 1% *n*-butanol, growth rate and final biomass concentration of both strains strongly declined and no growth was observed above 1.45% and 1.57% butanol for BY4741 and CEN.PK113-7D, respectively (Figure 2A and B). Strain BY4741 consumed all glucose within 48 h at *n*-butanol concentrations below 1.1%, the CEN.PK113-7D strain was able to consume all glucose up to a butanol concentration of 1.3%. Above these concentrations, residual glucose was still present after 48 h.

Based on these results, the OD_660_ observed after 48 h of incubation, can be used as a measure for *n*-butanol tolerance. To facilitate the genome-wide screening in the remainder of this manuscript, a Butanol Sensitivity Index (BSI) was calculated as the ratio of the optical density (48 h) of the culture grown in absence of *n*-butanol over the culture with 1% *n*-butanol.

$$\text{BSI}=\frac{\mathrm{OD}660\;\text{without}\;\text{butanol}}{\mathrm{OD}660\;\text{with}\;1\%\text{butanol}}$$

The reference strain BY4741 displayed a BSI value of 2. Strains that are more sensitive than this reference will have a BSI >2, while strains exhibiting a more tolerant phenotype with 1% *n*-butanol will have BSI values between 1 and 2.

### Screening for genes involved in *n*-butanol tolerance

To identify the molecular mechanisms involved in *n-*butanol tolerance in *S.cerevisiae*, the haploid nonessential gene deletion collection (5154 strains), made in the BY4741 genetic background [33] was screened for growth in the absence and presence of 1% *n*-butanol. The BSI values of the entire knockout collection were compared to BY4741, and no strain revealed increased tolerance compared to the reference. After multiple replicate tests (n=8), a total of 105 deletion strains were confirmed to be more sensitive towards *n*-butanol (Additional file 1: Table S1). The 55 strains displaying BSI values between 2 and 10 were to a large majority still able to grow in the presence of 1% *n*-butanol (final OD with 1% butanol > 0.1) although they reached a lower OD_660_ than the reference (Additional file 1: Table S1). The 50 strains exhibiting a BSI value above 10 (Additional file 1: Table S1) were unable to grow in the presence of 1% *n*-butanol (final OD with 1% butanol < 0.08), indicating a hyper-sensitive phenotype. To exclude specific effects of the BY4741 genetic background, gene deletions resulting in absence of growth at 1% *n*-butanol were reintroduced in a *S. cerevisiae* strain of the CEN.PK lineage, with the exception of dubious open reading frames (YKL118W, YPL062W, YBL094C, YLR338W and YDR157W). Additionally, *STP11* and *DID4* were arbitrarily choosen and tested in CEN.PK113-7D, bringing the total number of tested deletions to 47 (Additional file 1: Table S1). Interestingly, three deletions did not yield transformants (*GON7, UAF30* and *OCH1*). The genes *UAF30* and *GON7* have already been shown to be essential in the Sigma1278b strain [36]. No such obvious explanation was found for our inability to obtain an *och1Δ* strain in the CEN.PK genetic background.

**Figure 3:**
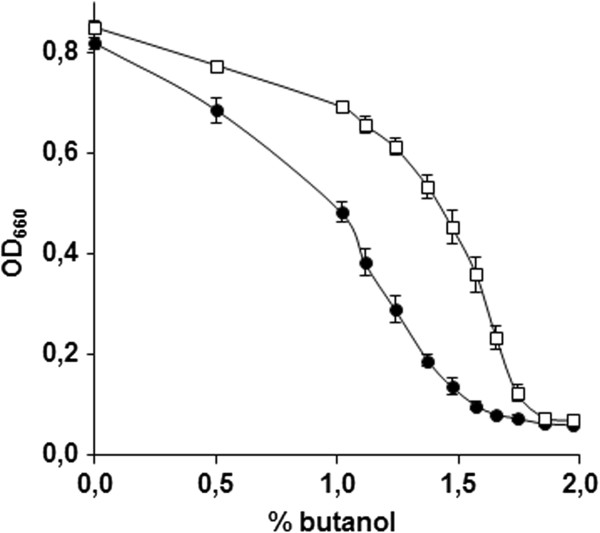
**An example of screening for *n*-butanol tolerance of a single deletion strain in the CEN.PK background, comparing IMK356 ( *pre9 *Δ) (●) and CEN.PK113-7D (□).** The strains were grown in 96 well plates in synthetic medium containing different concentrations of *n*-butanol, and the OD_660_ was measured after 48 h. Each point represents the average final OD_660_ and standard deviation for each *n*-butanol concentration, calculated from at least 16 independent cultures.

**Figure 4:**
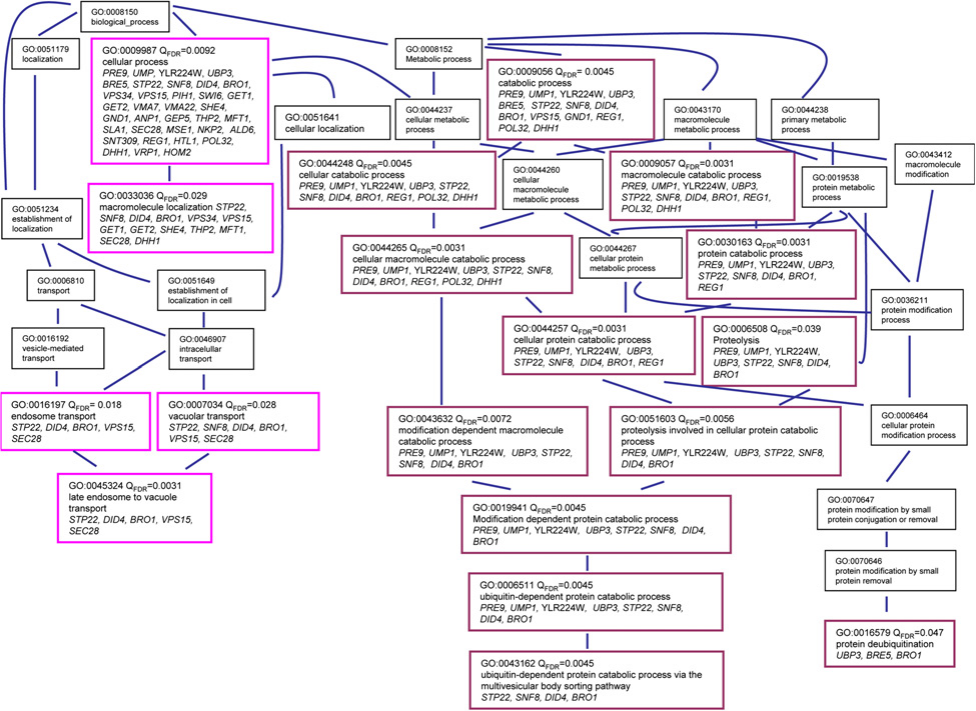
**Hierarchical map of the GO complete categories found enriched in the set of 35 genes whose deletion leads to higher *n*-butanol sensitivity phenotype in both BY4741 and CEN.PK113-7D strains.** Pink boxes denote enriched (Q_FDR_< 0.05) GO term categories related to formation of multivesicular bodies, and the purple boxes enriched (Q_FDR_< 0.05) GO term categories related to ubiquitin-proteasome system based on Fisher exact statistics.

Out the 44 viable deletion mutants, only 35 also showed an *n*-butanol sensitive phenotype in the CEN.PK background (Figure 3, Table 1). Fischer exact statistic based GO functional category enrichment analysis on these 35 genes revealed a significant enrichment of genes involved in protein-degradation processes, which could be separated in two groups: i) genes involved in the ubiquitin-proteasome system: *PRE9,* YLR224W, *BRE5, UBP3* and *UMP1*, and ii) genes involved in the formation of multivesicular bodies: *STP22*, *DID4*, *SNF8* and *BRO1* (Figure 4).

**Table 1:** **Set of 35 genes whose deletion results in *n*-butanol sensitivity in both BY4741 and CEN.PK113-7D genetic backgrounds**

**Gene**	**Function**
*PRE9*	Alpha 3 subunit of the 20S proteasome. *PRE9* encodes the only nonessential proteasome subunit.
*UMP1*	Chaperone required for correct maturation of the 20S proteasome.
YLR224W	Subunit of the SCF ubiquitin ligase complexes, responsible of recognizing misfolded proteins.
*UBP3*	Ubiquitin-specific protease and its cofactor, respectively. Responsible of the deubiquitination of proteins. Important to maintain the pull of free ubiquitin in the cells.
*BRE5*	
*STP22*	Components of the ESCRT-I, -II and –II complexes, respectively. Responsible of sorting ubiquitinated membrane proteins into Multivesicular Bodies (MVB) for their degradation in the vacuole.
*SNF8*	
*DID4*	
*BRO1*	Responsible of deubiquitination in the MBV. Important to maintain the pull of free ubiquitin.
*VPS34*	Vps34 and Vps15 form a complex responsible for the synthesis of phosphatidylinositol 3-phosphate, involved in endosomal membrane trafficking and in the regulation of protein sorting.
*VPS15*	
*PIH1*	Component of the conserved R2TP complex. Interacts with Hsp90 to mediate assembly large protein complexes such as box C/D snoRNPs and RNA polymerase II.
*SWI6*	Transcription cofactor required for the unfolded protein response.
*GET1*	Subunits of the GET complex. Involved in the insertion of tail anchored proteins into the ER. Tail anchored proteins play a role in vesicular traffic and folding or degradation of membrane proteins.
*GET2*	
*VMA7*	Subunit F of the eight-subunit V1 peripheral membrane domain of vacuolar H+−ATPase, and peripheral membrane protein that is required for vacuolar H+−ATPase function, respectively.
*VMA22*	
*SHE4*	Protein that regulates myosin function; involved in endocytosis.
*GND1*	6-phosphogluconate dehydrogenase, required for adaptation to oxidative stress.
*ANP1*	Subunit of the alpha-1,6 mannosyltransferase complex. Involved in osmotic sensitivity.
*GEP5*	Protein of unknown function, required for mitochondrial genome maintenance.
*THP2*	Subunits of the THO complex. Involved in transcription elongation by RNA polymerase II and in telomere maintenance.
*MFT1*	
*SLA1*	Cytoskeletal protein binding protein required for assembly of the cortical actin cytoskeleton.
*SEC28*	Epsilon-COP subunit of the coatomer; regulates retrograde Golgi-to-ER protein traffic.
*MSE1*	Mitochondrial glutamyl-tRNA synthetase.
*NKP2*	Non-essential kinetochore protein, subunit of the Ctf19 central kinetochore complex.
*ALD6*	Cytosolic aldehyde dehydrogenase, required for conversion of acetaldehyde to acetate.
*SNT309*	Member of the NineTeen Complex. Involved in splicing of nuclear RNAs via the spliceosome.
*REG1*	Regulatory subunit of Glc7p, involved in negative regulation of glucose-repressible genes.
*HTL1*	Component of the RSC chromatin remodeling complex. involved in telomere maintenance.
*POL32*	Third subunit of DNA polymerase delta, involved in chromosomal DNA replication.
*DHH1*	Cytoplasmic DExD/H-box helicase, stimulates mRNA decapping.
*VRP1*	Actin-associated protein involved in cytoskeletal organization and cytokinesis.
*HOM2*	Aspartic beta semi-aldehyde dehydrogenase, catalyzes the second step in the common pathway for methionine and threonine biosynthesis.

**Figure 5:**
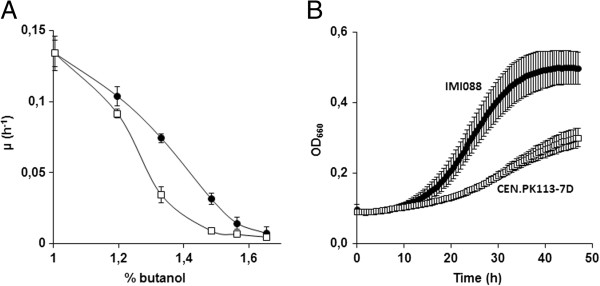
**Growth of the strains IMI088 (overexpressing YLR224W) (●) and CEN.PK113-7D (□) in the presence of *n-*butanol. A**: Growth rate in synthetic medium containing *n*-butanol concentrations ranging from 1% to 1.7%. The data represent the average apparent growth rate μ (h^-1^) and standard deviation of a minimum of four independent cultures. **B**: Growth in the presence of 1.33% *n*-butanol. The values correspond to the average final OD_660_ of three independent cultures and the standard deviation of replicate cultures.

To investigate whether the 35 genes that conferred sensitivity upon deletion also confer resistance to *n*-butanol upon overexpression, the native promoters of their chromosomal *loci* in CEN.PK113-7D were systematically replaced by the strong constitutive *TPI1* promoter. Subsequently the overexpression strains were tested for butanol tolerance/sensitivity phenotype. Of the 35 overexpression strains, only one displayed a more tolerant phenotype while the *n*-butanol tolerance of the other 34 was similar to that of the reference strain CEN.PK113-7D. The overexpression of YLR224W, encoding the subunit of a Skp-Cullin-F-box (SCF) ubiquitin ligase complex responsible for the recognition of damaged proteins [37,38], did result in a strain with an increased *n*-butanol tolerance (Figure 5). Up to a concentration of 1% *n*-butanol, the specific growth rate and biomass yield of strain IMI088 (overexpressing YLR224W) was very similar to CEN.PK113-7D. At higher concentrations, IMI088 was significantly more tolerant (Figure 5A). In the presence of 1.33% butanol, the growth rate of IMI088 was 0.074 h^-1^, twofold higher than that of the reference strain CEN.PK113-7D at this *n*-butanol concentration, and it showed a higher biomass yield (Figure 5B). Moreover CEN.PK113-7D barely grew at *n*-butanol concentrations above 1.48%, while IMI088 could grow in the presence of concentrations as high as 1.75% *n*-butanol. This indicated that overexpression of YLR224W did not only lead to an increase in growth rate and biomass yield in the presence of *n*-butanol concentrations permissive to the reference strain, but also increased the maximum *n*-butanol concentration at which yeast cells can grow.

### Evolutionary engineering for increased butanol tolerance

**Figure 6:**
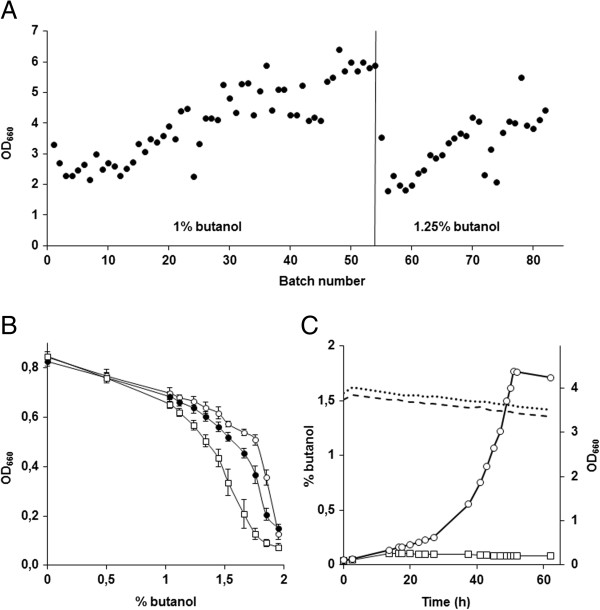
**Evolutionary engineering of *n*-butanol tolerance in sequential shake flask cultivation. A**: OD_660_ measured at the end of each batch throughout the evolution process. After 55 batches (vertical line) the *n*-butanol concentration was raised to 1.25%. The laboratory evolution was stopped after 83 batches. **B**: *n*-Butanol tolerance of the evolved strains IMS0351 (○) and IMS0344 (●) and the reference strain CEN.PK113-7D (□). The strains were grown in 96 well plates in synthetic medium in the presence of *n*-butanol concentrations ranged from 0 to 1.9%. The data represent the average and the standard deviation of the biomass yield (OD_660_) measured after 48 h from 16 independent cultures. **C**: Growth (OD_660_) in anaerobic pH controlled-bioreactor of the strains IMS0351 (○) and CEN.PK113-7D (□) in the presence of 1.5% *n*-butanol. The concentration of *n*-butanol throughout the experiments is shown for IMS0351 (----) and CEN.PK113-7D (**····**).

To complement the screening of the mutant library, we applied laboratory evolution and analysis of the resulting evolved strains [35,39-41]. To select for spontaneous mutants with improved tolerance based essentially on specific growth rate and biomass yield [42], two independent evolution lineages of CEN.PK113-7D were started in shake flasks containing synthetic medium with 1% *n*-butanol. The shake flasks were sealed to prevent butanol evaporation, thereby keeping the selective pressure constant throughout the experiment. When stationary phase was reached, OD_660_ was measured and a new shake flask was inoculated from the previous one. In the first evolution line, the biomass concentration reached at stationary phase had increased by more than 2-fold relative to first cultures after 55 serial transfers (Figure 6A). The *n*-butanol concentration was further increased to 1.25% and kept constant for an additional 28 transfers. A single-cell line that could grow as fast (±10%) as the evolved population at a *n*-butanol concentration of 1.46% was isolated and named IMS0344. In the second evolution experiment, the *n*-butanol concentration was increased to 1.2% after 30 transfers, to 1.3% after 42 transfers and to 1.35% after 52 transfers. After the 63^rd^ shake flask, a single-cell line was isolated and named IMS0351. The evolved strains IMS0344 and IMS0351 showed increased butanol tolerance compared to CEN.PK113-7D (Figure 6B). This difference was especially significant in presence of *n*-butanol concentration above 1.48%, where only IMS0344 and IMS0351 could grow. At a high *n*-butanol concentration of 1.75%, IMS0344 and IMS0351 displayed stationary-phase OD_600_ values that were 25% and 50% lower, respectively, that found in cultures without butanol and they both retained growth up to a butanol concentration of 1.85%.

To further characterize the evolved mutants, we tested and compared the growth of the most tolerant strain, IMS0351, with that of the parental strain CEN.PK113-7D in anaerobic bioreactor cultures grown on synthetic medium with 1.5% *n*-butanol. Under these conditions, CEN.PK113-7D was unable to grow, while IMS0351 exhibited a growth rate of 0.085 h^-1^ (Figure 6C), thereby confirming the increased tolerance of the evolved strain. Although the *n*-butanol concentration decreased slightly due to the nitrogen sparging in these cutlures, the butanol concentration remained above 1.38% throughout the fermentation (Figure 6C).

### Tolerance of the evolved strains to different alcohols

**Figure 7:**
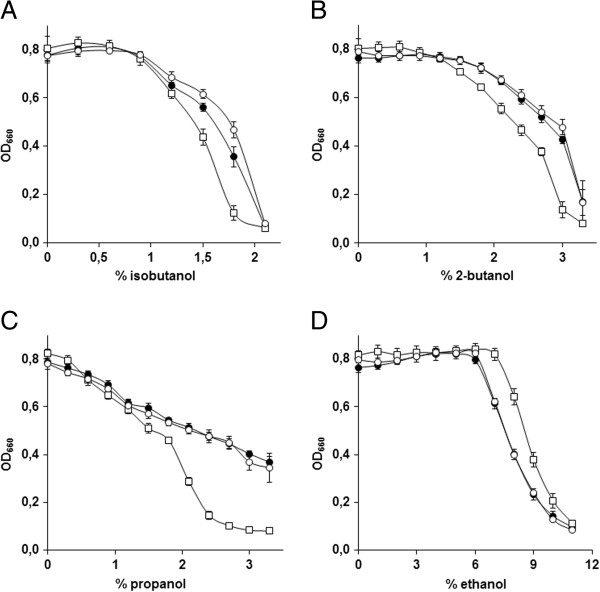
**Tolerance of the strains IMS0344 (●) and IMS0351 (○) and CEN.PK113-7D (□) to different alcohols.** The strains were grown in 96 well plates containing synthetic medium with increasing concentrations of isobutanol, 2-butanol, propanol and ethanol. The strains were grown in 96 well plates in synthetic medium in the presence of different concentration of alcohols. The data represent the average and the standard deviation of the biomass yield (OD_660_) measured after 48 h from 16 independent cultures. **A**: Growth in the presence of isobutanol, **B**: Growth in the presence of 2-butanol, **C**: Growth in the presence of propanol, **D**: Growth in the presence of ethanol.

*n*-butanol is one of the four C4-monoalcohols, along with *tert*-butanol, *sec*-butanol, and isobutanol. Although all four butanol isoforms might be used, isobutanol has especially attractive physical properties for use as a biofuel (i.e. high octane number and low melting temperature [41]). To investigate whether the evolved *n*-butanol-tolerant strains were also more tolerant to other short-chain monoalcohols, we tested their growth in the presence of butanol isomers, propanol and ethanol. Strains IMS0344, IMS0351 and CEN.PK113-7D were grown in 96-well plates containing synthetic medium with different concentrations of the alcohols, and OD_660_ was measured after 48 h of incubation. All alcohols tested inhibited growth, although the concentration ranges at which inhibition was observed differed. The toxicity of isobutanol was very similar to that of *n*-butanol and none of the strains grew at concentrations higher than 2% (Figure 6B, Figure 7A). The evolved *n*-butanol-tolerant strains showed a markedly increased tolerance to isobutanol. In the presence of 1.8% isobutanol, strains IMS0344 and IMS0351 reached stationary-phase OD_660_ values that were 4-fold and 6-fold higher, respectively, than observed with the parental strain CEN.PK113-7D. The tolerance of IMS0351 and CEN.PK113-7D was further tested in anaerobic bioreactor cultures grown on synthetic medium with 1.2% isobutanol. Under these conditions, the evolved strain IMS0351 grew at a specific growth rate of 0.13 h^-1^, while CEN.PK113-7D only grew at 0.07 h^-1^. Furthermore, the OD_660_ reached at the stationary phase was also two fold higher for IMS0351. The effect of 2-butanol on the cells was less severe than that of *n*-butanol and isobutanol, and CEN.PK113-7D could grow up to a concentration of 3%. The evolved *n*-butanol tolerant strains were also more tolerant to 2-butanol and, in the presence of 3% 2-butanol, stationary-phase OD_660_ of IMS0344 and IMS0351 were 3-fold and 3.5-fold higher, respectively than that of the parental strain (Figure 7B). The differences in tolerance to propanol between the evolved *n*-butanol-tolerant strains and the reference strains were larger than for the butanol isomers. In the presence of 2.4% propanol, growth of the parental strain CEN.PK113-7D was strongly impeded while both evolved strains could grow in the presence of 3.3% propanol, where they reached almost 50% of the stationary-phase OD_660_ observed under non-stressed conditions (Figure 7C). Interestingly, the evolved strains showed a lower tolerance to ethanol than the reference strain (Figure 7D), indicating different inhibition and/or tolerance mechanisms for C3 and C4 alcohols than for C2 alcohols.

### Identification and reverse engineering of the mutations present in evolved butanol-tolerant strains

**Figure 8:**
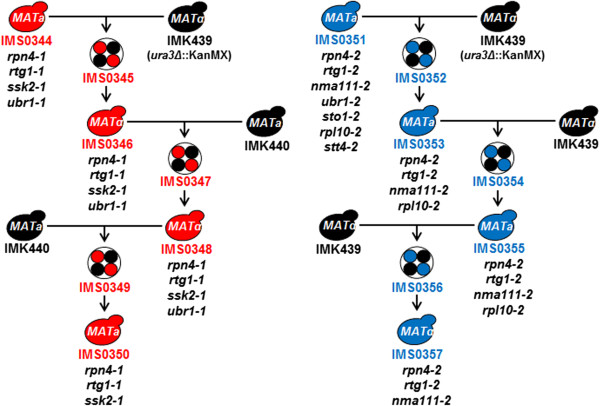
**Segregation of the mutations found in the evolved *n*-butanol tolerant strains IMS0344 and IMS0351 and the F1, F2 and F3 generation of back crossing.** The evolved strains IMS0344 and IMS0351 were crossed with IMK439 (*MATα* isogenic of CEN.PK113-7D and deleted in *URA3*) to create a diploid. The diploid was sporulated, one haploid segregant with the same tolerance as the evolved strain was selected and the cycle repeated was repeated three times.

**Figure 9:**
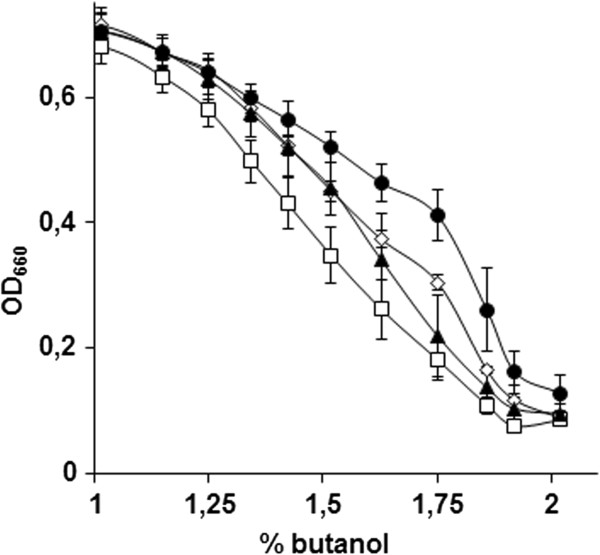
***n*-Butanol tolerance of the strains IMI218 (containing the *rpn4-1 *allele) (▲), IMI238 (containing the *rtg1-1 *allele) (◊), CEN.PK113-7D (□) and the evolved strain IMS0344 (●).** The data represent the average and the standard deviation of the biomass yield (OD_660_) measured after 48 h from at least 16 independent cultures in presence of different concentration of *n*-butanol.

To study the molecular basis of increased butanol tolerance, the genomes of the strains IMS0344 and IMS0351 were sequenced and compared to the reference CEN.PK113-7D genome [43]. Both strains were sequenced with very high coverage allowing high quality mapping. Mapping analysis of the raw sequence data of strain IMS0344 on the CEN.PK 113-7D reference genome identified four single-nucleotide differences within open reading frames that resulted in an amino acid change or in the introduction of an early stop codon. In the case of the strain IMS0351, five single-nucleotide variations were found within open reading frames and two additional mutations outside open reading frames (Figure 8, Table 2). Interestingly, three genes (*RPN4*, *RTG1* and *UBR1)* were independently and differently mutated in both evolution lineages (Table 2). To assess whether and to what extent these mutations contributed to butanol tolerance, the evolved strain IMS0344 was crossed with a *Matα ura3Δ* strain (IMK439) isogenic to the ancestor strain CEN.PK113-7D (Figure 8). The resulting diploid (IMS0345) was sporulated and haploid segregants were screened for *n*-butanol tolerance. A haploid strain of the 1^st^ generation (F1) exhibiting the same *n*-butanol tolerance as the evolved strain was selected (strain IMS0346). Two additional backcrossing cycles with strain IMK440 (*Mata ura3Δ*) were performed to generate butanol-tolerant haploid strains of second (F2) (IMS0348) and third (F3) generation (IMS0350) (Figure 8). Strain IMS0351 isolated from the second evolution line was similarly backcrossed with IMK439, yielding the butanol tolerant haploids IMS0353 (F1), IMS0355 (F2) and IMS0357 (F3), respectively (Figure 8). All F1, F2 and F3 butanol-tolerant haploids were sequenced. In the F3 strain IMS0350, the allele *ubr1-1* was absent; indicating that is was not required for butanol tolerance. In the other F3 strain, IMS0357, only three mutated alleles remained, *rpn4-2, rtg1-2 and nma111-2*, indicating that *ubr1-2*, *sto1-2, rpl10-2* and *stt4-2* were not required for butanol tolerance. Interestingly, mutated alleles of both *RPN4* and *RTG1* were found in the F3 haploid, butanol-tolerant segregants of the two independent evolution lines, strongly suggesting that they contributed to the butanol tolerant phenotype (Table 2). To test this hypothesis, we reversed engineered the alleles *rpn4-1* and *rtg1-1* in CEN.PK113-7D. The butanol tolerance of the resulting strains was tested and compared with both IMS0344 and CEN.PK113-7D (Figure 9). The constructed strains were more tolerant than CEN.PK113-7D, thereby confirming the relevance of the genes *RPN4* encoding a transcription factor that stimulates expression of proteasome genes and *RTG1* encoding a transcription factor involved in interorganelle communication for butanol tolerance. However, none of the strains was as tolerant as IMS0344, suggesting a synergistic effect of both mutated alleles to reach the tolerance level of the evolved strain.

**Table 2:** Single nucleotide variation identified in the evolved strains IMS0344 and IMS0351 by whole-genome resequencing

**Strain**	**Mutated allele**	**Nucleotide change**	**Amino acid change**
IMS0344	*rpn4-1*	G1518C	K506N
	*rtg1-1*	A235G	K79E
	*ssk2-1*	C3974A	P1325Q
	*ubr1-1*	A3194G	E1065G
IMS0351	*rpn4-2*	C1546T	Q516*
	*rtg1-2*	C256A	L86I
	*nma111-2*	C545A	S182*
	*ubr1-2*	C2129A	S710*
	*sto1-2*	T2543G	F736V
	*rpl10-2*	change from G to C 296 bp upstream of *RPL10*,	——————
	*stt4-2*	change from T to C 984 bp upstream of *SST4*.	——————

* denotes the introduction of an early stop codon.

## Discussion

**Figure 10:**
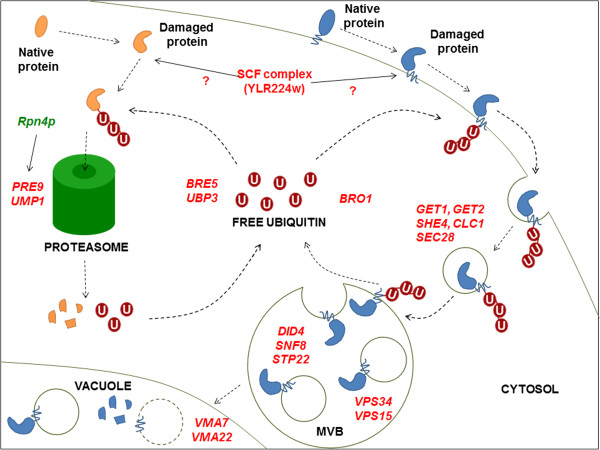
**Protein degradation under butanol stress.** The gene deletion that resulted in strains with a reduced butanol tolerance are shown in red. After their ubiquitination, membrane proteins are internalized via endocytosis and endocytic vesicles fuse with the membrane of Multivesicular Bodies (MBVs). In the MBVs, proteins are deubiquitinated and sorted into vesicules. When the membrane of the MBVs fuses with the vacuole, releases the vesicles that are degraded by vacuolar hydrolases. Ubiquitinated cytosolic proteins are degraded in the proteasome, producing small peptides and free ubiquitin. Rpn4p induces the expression of proteasome genes including *PRE9*, and was found to be relevant for butanol tolerance in the evolutionary engineering approach.

Both the screening of the *S. cerevisiae* haploid deletion collection and analysis of evolved mutants with improved *n*-butanol tolerance, indicated that protein turnover plays a key role in butanol tolerance. Genes whose deletion led to increased *n*-butanol sensitivity showed an overrepresentation of functions related to protein degradation via the ubiquitin-proteasome and vacuole (Figure 10). More specifically, the products of these genes included a protein involved in maturation of the 20S proteasome (Ump1) [44-47], the α3 subunit of the 20S proteasome (Pre9) [44-47], a sub-unit of the SCF-ubiquitin ligase complex (Ylr224W), deubiquitin proteases (Bre5 and Ubr3), sub-units of the ESCRT machinery (Bro1, Did4, Snf8, Spt22) [48-52], proteins involved in cellular trafficking, protein sorting and endocytosis (Vps15, Vps34, Get1, Get2, She4, Clc1, Sec28) [53-58] and transcriptional regulators of the proteasome and the unfolded protein response (Rpn4, Swi6) (Table 1, Figures 4 and 10). Some mutations identified during the screen of the deletion collection were previously found to confer sensitivity to ethanol, 1-propanol and 1-pentanol (*bro1Δ, pre9Δ, spt22Δ*, *ump1Δ* and *she4Δ)*[59,60], suggesting that protein degradation plays a more general role under alcohol stress conditions.

The only gene identified in the screening of the deletion library whose overexpression led to increased butanol tolerance was YLR224W, which encodes one of 22 F-box proteins in *S. cerevisiae*[61] (http://www.yeastgenome.org/). Together with Skp1, Cdc53 and Rbx1, individual F-box proteins form SCF ubiquitin-ligase complexes involved in ubiquitin-dependent protein degradation. F-box proteins contribute to the specificity of SCF complexes by, independently of the complex, aggregating to target proteins and subsequently binding to Skp1 [37,62]. With the exception of Met30 and Cdc4, all *S. cerevisiae* F-box protein deletion mutants are viable. However, in our screen, YLR224W was the only gene encoding an F-box protein whose deletion led to increased butanol sensitivity. This suggests that Ylr224W specifically targets a (subset of) protein(s) whose degradation is important for butanol tolerance. A similar conclusion was drawn based on a previous observation that overexpression of YLR224W conferred increased tolerance to methylmercury [38]. Overexpression of genes whose products are involved in proteasome-driven protein degradation, but act downstream of YLR224W, did not result in improved tolerance (Figure 10). This suggested that ubiquitination of YLR224W targets is the rate-limiting step in their degradation. Consistent with this notion, the effect of the overexpression of YLR224W on methylmercury tolerance was suppressed by the proteasome inhibitor MG132. [38]. Identification of the protein targets of Ylr224W should provide additional insight in the molecular mechanisms of butanol sensitivity and tolerance in *S. cerevisiae.*

Screening of the haploid deletion collection of *S. cerevisiae* was limited to an exploration of the impact of loss-of-function mutations in non-essential genes on butanol tolerance. The laboratory evolution strategy allowed inspection of a much wider genetic landscape, which was not limited to non-essential genes but also widely expanding the type of mutations (i.e. nucleotide variation, small insertion-deletion and duplication [35]) resulting in proteins with modified catalytic or structural activities. This approach is especially relevant for the exploration of central cellular processes such as protein degradation, in which a substantial fraction of the genes involved cannot be studied via haploid deletion strains because their protein products are essential for growth.

In two independently evolved butanol-tolerant strains, mutations in *RPN4* and *RTG1* were shown to contribute to increased butanol tolerance. *RPN4* encodes a transcription factor involved in the expression of proteasome genes [63] and is required for normal levels of intracellular proteolysis. Rpn4 is an unstable protein characterized by an extremely short half life [64]. It is regulated by a negative feedback circuit; Rpn4 promotes expression of proteasome genes and is itself subject to proteasomal degradation [65]. The distinct *RPN4* alleles present in the two evolution lines might, for example, encode Rpn4 versions that are less susceptible to degradation by the proteasome or that are more active transcription factors. As a result of this change, the transcription of proteasome genes would be increased or maintained in time, thus increasing or extending the activity of the proteasome. Such a mechanism would be entirely consistent with the important role of protein degradation in butanol tolerance revealed by the screening of yeast deletion mutants.

*RTG1* encodes a transcription factor involved in the communication between mitochondria and the nucleus [66] and it is also required for the expression of genes encoding peroxisomal proteins [67]. Reverse engineering of the *rtg1-1* allele in CEN.PK113-7D resulted in a strain with an increased butanol tolerance. Additionally, the strain defective in *RTG1* in the knockout collection showed an increased butanol sensitivity relative to BY4741 (Additional file 1: Table S1), indicating its relevance for tolerance. However, the mechanism by which the mutated alleles present in the evolved strains confer tolerance is unclear and will necessitate further dedicated experimental work.

This study has identified several defined mutations that can be applied to increase tolerance to propanol and butanol isomers in *S. cerevisiae*. While the resulting significant increases in tolerance are modest from the viewpoint of industrial application, the methodology used in this study should be directly applicable for further improvement of tolerance to these and other compounds. In particular, this study demonstrates how combining whole-genome sequencing and classical yeast genetics can accelerate the analysis and reverse engineering of strains generated by laboratory evolution. Over the past few years, molecular characterization of evolutionary engineered yeast strains has been strongly facilitated by the fast developments in Next Generation Sequencing methodologies [35,40,41,68]. However, establishing which of the observed mutations contribute to the selected phenotype still remained a laborious process, in which mutations have to be reversed engineered in a naive strain, either individually or in combination. Several solutions have been proposed to simplify the detection of biologically relevant mutations. First, availability of a high-quality reference genome sequence for the parental strain greatly contributes to reducing the number of false positives resulting from sequence comparison with evolved strains [43]. In this study, direct comparison of the evolved strains to the assembled genome of the non-evolved parent led to fewer than 10 mutation calls in each evolutionary run. Secondly, the use of independent, parallel evolution lines may rapidly identify genes or processes that are affected by mutation in independent experiments and are therefore likely to contribute to the observed phenotype [35,40,69]. In this study, we used backcrossing, a classical approach in yeast genetics, to enrich and isolate mutations that contributed to butanol tolerance in evolved strains. The tremendous power of this approach is evident from the fact that, after only three cycles of back crossing, the selected F3 segregant IMS0344 retained only three mutations of which, after reverse engineering, two were shown to directly contribute to butanol tolerance of the original evolved strain.

## Conclusion

Screening of a deletion mutant library of *S. cerevisiae* and analysis of butanol-tolerant strains isolated by whole genome sequencing revealed that protein degradation is a key process in the tolerance of *S. cerevisiae* to *n*-butanol. Mutations in three genes were shown to significantly increase butanol tolerance and can be applied in metabolic engineering strategies for butanol production with this yeast. Combination of whole-genome sequencing of evolved strains with repeated backcrossing to a native, isogenic strain was shown to be an extremely powerful approach to identify biologically relevant mutations. Further studies on the molecular mechanisms by which protein turnover affects butanol tolerance should provide further leads for strain improvement.

## Methods

### Strains, media and growth conditions

The *Saccharomyces cerevisiae* strains used in this study are listed in Table 3. The reference strains used in the study are BY4741 [70] and CEN.PK113-7D [43,71,72]. *S.cerevisiae* strains were routinely grown in YPD medium (yeast extract 10 g/l, peptone 20 g/l, glucose 20 g/l), and in synthetic medium as previously described in [73] but with the following modification: ammonium sulphate (5 g/l) was replaced by 2.3 g/l urea as the sole nitrogen source. The butanol tolerance tests and evolution in the presence of butanol were done in synthetic medium. Butanol was used at concentrations ranging from 0% to 1.9% (always expressed as v/v). The selection of strains transformed with the KanMX marker was done in solid YPD medium (containing 20 g/l agar) supplemented with G418 (InvivoGen, San Diego, CA) at a concentration of 200 μg/ml.

**Table 3:** ***Saccharomyces cerevisiae *strains used in this study**

**Strain name**	**Description**	**Source**
BY4741	*MAT*a *his*3Δ1 *leu*2Δ *met*15Δ *ura*3Δ	Euroscarf^a^
Yeast KO collection	*MAT*a *his*3Δ1 *leu*2Δ *met*15Δ *ura*3Δ ORFΔ::KanMX	OpenBiosystems^b^
CEN.PK113-7D	*MATa*	Euroscarf^a^
CEN.PK113-1A	*MATα*	Euroscarf^a^
IMK439	*MATα ura3Δ*::*KanMX*	This study
IMK440	*MATa ura3Δ*::*KanMX*	This study
Deletion strains*	*MATa ORFΔ::KanMX*	This study
Overexpression strains*	*MATa ORF*_pr_::*KanMX*-*TPI1*_pr_::ORF	This study
IMS0344	*MATa rpn4-1 rtg1-1 ubr1-1 ssk2-1*	This study
IMS0345	*MATa*/*MATα RPN4/rpn4-1 RTG1/rtg1-1 UBR1/ubr1-1 SSK2/ssk2-1 URA3/ura3Δ*::KanMX	This study
IMS0346	*MATα rpn4-1 rtg1-1 ubr1-1 ssk2-1*	This study
IMS0347	*MATa*/*MATα RPN4/rpn4-1 RTG1/rtg1-1 UBR1/ubr1-1 SSK2/ssk2-1 URA3/ura3Δ*::KanMX	This study
IMS0348	*MATα rpn4-1 rtg1-1 ubr1-1 ssk2-1*	This study
IMS0349	*MATa*/*MATα RPN4/rpn4-1 RTG1/rtg1-1 UBR1/ubr1-1 SSK2/ssk2-1 URA3/ura3Δ*::KanMX	This study
IMS0350	*MATa rpn4-1 rtg1-1 ssk2-1*	This study
IMS0351	*MATa rpn4-2 rtg1-2 ubr1-2 nma111-2 rpl10-2 sto1-2 sst4-2*	This study
IMS0352	*MATa*/*MATα RPN4/rpn4-2 RTG1/rtg1-2 UBR1/ubr1-2 NMA111/nma111-2 RPL10/rpl10-2 STO1/sto1-2 SST4/sst4-2 URA3/ura3Δ*::KanMX	This study
IMS0353	*MATa rpn4-2 rtg1-2 nma111-2 rpl10-2*	This study
IMS0354	*MATa*/*MATα RPN4/rpn4-1 RTG1/rtgi-2 NMA111/nma111-2 RPL10/rpl10-2 URA3/ura3Δ*::KanMX	This study
IMS0355	*MATa rpn4-2 rtg1-2 nma111-2 rpl10-2*	This study
IMS0356	*MATa*/*MATα RPN4/rpn4-2 RTG1/rtg1-2 NMA111/nma111-2 RPL10/rpl10-2 URA3/ura3Δ*::KanMX	This study
IMS0357	*MATa rpn4-2 rtg1-2 nma111-2*	This study
IMI218	*MATa RPN4*_pr_::KanMX *RPN4*_pr_*-rpn4-1*	This study
IMI238	*MATa RTG1*_pr_::KanMX *RTG1*_pr_*-rtg1-1*	This study

* the complete list of the deletion and overexpression strains is provided in Additional file 1: Table S1. ^a^http://web.uni-frankfurt.de/fb15/mikro/euroscarf/, ^b^http://www.thermoscientificbio.com/molecular-biology/.

The crossing studies were done in solid synthetic medium [73] (containing 20 g/l agar) without uracil and supplemented with G418 (InvivoGen) at a concentration of 200 μg/ml. For sporulation of diploid strains, they were grown in YPA (yeast extract 10 g/l, peptone 20 g/l, potassium acetate 10 g/l) and transferred to the sporulation medium (20 g/l potassium acetate, pH 7.0) [74].

### Screening of the deletion collection

The collection was propagated in YPD 24 hours at 30°C. Per strain 5μl of YPD grown cells were inoculated in flat bottom 96 well plates (one strain per well) (cat N^o^: 655161 Greiner bio-one, Alphen aan den Rijn, The Netherlands) containing 200 μl of synthetic medium, in the presence and absence of 1% butanol. The plates were sealed with a gas impermeable tape (cat N^o^: 236366 NUNC, Roskilde, Denmark) and incubated at 30°C for 48 hours without shaking. After the incubation time, the cells were resuspended in an orbital shaker (MS3, IKA-Werke GmbH & Co, Staufen, Germany). The sealing tape was removed and the optical density of each well was measured in a GENIos Pro microplate spectrophotometer (Tecan, Männedorf, Switzerland) at a wavelength of 660 nm. The measured OD_660_ values were used to calculate the Butanol Sensitivity Index (BSI) that is defined as follows:

$$\text{BSI}=\frac{\mathrm{OD}660\;\text{without}\;\text{butanol}}{\mathrm{OD}660\;\text{with}\;1\%\text{butanol}}$$

### Analysis of butanol tolerance in 96 well plates

The strains were grown in YPD for 24 hours at 30°C and 200 rpm. The OD_660_ of the cultures was measured and they were diluted to an OD_660_ of 5 with fresh YPD. 96 well plates were filled with 200 μl/well of synthetic medium containing butanol concentrations ranging from 0 to 1.9% (12 different concentrations, 8 replicas per condition). The plates containing synthetic medium with butanol were inoculated from the diluted YPD cultures with the use of a 96 well pin replicator. The plates were sealed with a gas impermeable tape (NUNC) and incubated at 30°C for 48 hours without shaking. After the incubation time, the cells were resuspended in an orbital shaker (MS3, IKA-Werke GmbH & Co), the sealing tape was removed and the OD_660_ of each well was measured in a GENIos Pro microplate spectrophotometer (Tecan). At least 2 plates were inoculated per strain and for each butanol concentration the average OD_660_ was calculated with the data from both plates (16 replicas).

A 96 well plate setup was also used to compare the growth of two different strains in the presence of increasing concentrations of butanol. The strains were grown in YPD for 24 hours at 30°C and 200 rpm. From these cultures, 12 aliquots of 1 ml of synthetic medium with butanol concentrations ranging from 0% to 1.9% (12 different concentrations) were inoculated at an OD_660_ of 0.1. Each inoculated aliquot was distributed in 4 wells of a 96 well plate (200 μl per well). The plates were sealed with a gas impermeable sealing tape (NUNC) and incubated at 30°C in a GENIos Pro microplate spectrophotometer (Tecan) for 48 hours. A measurement of the OD_660_ of each well was taken every 30 minutes.

### Anaerobic serial transfer for evolutionary engineering

Two independent sequential batch cultures of the strain CEN.PK113-7D were cultivated in synthetic medium [73] containing 1% of butanol. The cultivation was carried out in shake flask, using closed bottles to prevent butanol evaporation. After each batch, the OD_660_ of the cultures was measured and a new shake flask was inoculated from the previous one to an OD_660_ of 0.1. When a higher final OD_660_ was observed, the butanol concentration of the following batch was increased. In the first evolution line the butanol concentration was increased up to 1.25% after 55 batches. After 83 batches a colony was isolated and its butanol tolerance tested. In the second evolution line the butanol concentration was increased up to 1.2% after 30 batches, to 1.3% after 42 batches and to 1.35% after 52 batches. After 63 batches a colony was isolated and its butanol tolerance tested. The strains evolved for butanol tolerance in the first and second cultures are IMS0351 and IMS0351, respectively.

### Batch fermentation in bioreactor in the presence of butanol and isobutanol

Each strain was grown for 24 hours in synthetic medium containing 2.3 g/l urea as the sole nitrogen source and 1% *n*-butanol or isobutanol, respectively. Cultivation was performed in 2 l bioreactors (Applikon, Schiedam, The Netherlands) containing 1 l of synthetic medium with 20 g/l glucose, and supplemented with 0.01 g/l ergosterol and 0.42 g/l Tween 80 for anaerobic cultivation. Antifoam Emulsion C (Sigma-Aldrich, Zwijndrecht, The Netherlands) was prepared as a 20% (w/v) solution, sterilized and added into the bioreactor at a final concentration of 0.2 g/l. The bioreactors were inoculated at an OD_600_ of 0.1 and after inoculation; the right concentration of *n*-butanol or isobutanol was added. Cultures were stirred at 800 rpm and kept anaerobic by flowing N_2_ gas in the head space at a flow-rate of 0.1 l/min. The pH of the culture was kept at 5 by automated addition of 2.0 M KOH. Samples were taken over time to measure the OD of the cultures and determine butanol and glucose concentrations. Glucose, ethanol, glycerol and butanol concentrations were analysed via HPLC using an Aminex HPX-87H ion exchange column operated at 60°C with 5 mM H_2_SO_4_ as mobile phase at a flow rate of 0.6 ml/min.

### Construction of the deletion and overexpression strains

In order to delete the target genes in CEN.PK113-7D, the deletion cassettes were prepared by direct amplification of the deleted gene in the corresponding strain from the BY knockout collection (Additional file 1: Table S2). The genomic DNA of the strains from the collection was extracted with the YeaStar™ Genomic DNA kit (Zymo Research, Irvine, CA) following the instructions of the manufacturer, and used as a template to amplify deletion cassettes. The deletion cassettes were amplified with the Phusion high-fidelity DNA polymerase (Thermo Scientific, Landsmeer, The Netherlands), with primers annealing at least 100 base pairs upstream and downstream of the KanMX marker, corresponding to the promoter and terminator regions of the genes, respectively (Additional file 1: Table S3). The deletion cassettes were transformed in CEN.PK113-7D by the lithium acetate method [75], inserted in the genome by homologous recombination and the selection of transformants was done in YPD plates containing 200 μg/ml G418. The confirmation of the deletion was done by PCR amplification with DreamTaq DNA Polymerase (Thermo Scientific), with a forward primer that anneals upstream of the insertion point and the reverse primer KanMX-DCR that anneals in the KanMX gene. In some cases, the confirmation was done with the forward primer KanMX-DCF (annealing in the KanMX gene) and a reverse primer annealing downstream of the insertion point. The amplification with these two primer pairs is only possible if the ORF of the gene has been successfully replaced by KanMX.

The overexpression of the genes was done by inserting upstream of their ORF the strong constitutive promoter from the gene *TPI1*. Overexpression cassettes containing the KanMX marker and the *TPI1* promoter were amplified from the plasmid *pUG6-TPI1 prom*, with the Phusion high-fidelity DNA polymerase (Thermo Scientific) and the primer pair named with the suffixes –OF and –OR, stating for overexpression forward and reverse, respectively (Additional file 1: Table S4). The primers –OF contain 50 base pairs homologous to the promoter region of each gene and the –OR ones, homologous to the beginning of the ORF. The overexpression cassettes were transformed in CEN.PK113-7D by the lithium acetate method [75], inserted in the genome by homologous recombination and the selection of transformants was done in YPD plates containing G418. The confirmation of the insertion was done by PCR amplification DreamTaq DNA Polymerase (Thermo Scientific) and the forward primer TPI1prom-ICF, annealing in the promoter of *TPI1* and the reverse primer named with the suffix –OCR, annealing in the ORF of each gene. The amplification with these two primers is only possible if the promoter from *TPI1* has been successfully inserted upstream of the ORF. In both the confirmation of the deletion and overexpression of the genes, a negative control PCR was done with the primer pair –OCF and –DCR that amplifies only in the native allele.

### Reverse engineering of the mutated *rpn4-1* and *rtg1-1* alleles

The gDNA of the strain IMS0344 was extracted with the YeaStar™ Genomic DNA kit (Zymo Research) following the instructions of the manufacturer, and used as a template to amplify by PCR the alleles *rpn4-1* and *rtg1-1*. For the reverse engineering of the mutated alleles in CEN.PK113-7D, two different cassettes were used. One cassette consists of the marker gene KanMX, flanked in one side by 50 base pairs homologous to a region upstream of the target gene, and in the other side by a *s*ynthetic sequence to promote homologous *r*ecombination (SHR). The second cassette consists of the mutated gene flanked in one side by the same SHR sequence used in the first cassette (Additional file 1: Table S5). The primers used for the amplification of the cassettes and for the analysis of the transformants are listed in Additional file 1: Table S6. When cotransforming both cassettes into CEN.PK113-7D, they recombine through the SHR sequence creating one single cassette. The new cassette is inserted into the corresponding locus in the genome by homologous recombination, replacing the original allele by the mutated one. The insertion of the cassette in the right locus was checked by PCR (Additional files 1: Tables S5 and S6), where a PCR product is only obtained if the cassette has been successfully inserted. The replacement of the original allele by the mutated one was checked by Real Time-PCR coupled with a High Resolution Melting analysis of the amplified product. The primers amplify a fragment of a size between 80 to 200 base pairs that includes the region with the SNV. The PCR mix was made by using the Type-it^®^ HRM™ kit following the instructions of the manufacturer and the amplification was done into a Rotor-Gene Q thermocycler (Qiagen, Venlo, The Netherlands). After the amplification step, the temperature was increased from 65°C to 95°C in steps of 0.1°C and the fluorescence was recorded. The fluorescence profile of the samples was compared with the ones from IMS0344 (positive control) and CEN.PK113-7D (negative control).

### Crossing, sporulation and screening for butanol tolerance of the haploid segregants

Two ancestor strains with different mating types were used for the crossing; IMK439 (*MATa*) and IMK440 (*MATα*), derived from CEN.PK113-7D and CEN.PK113-1A respectively, in which the *URA3 locus* has been replaced by the KanMX marker. The cassettes for the deletion of *URA3* was amplified from the plasmid *pUG6-TPI1 prom* with the primers URA3-KanMXF and URA3-KanMXR, and transformed into CEN.PK113-7D and CEN.PK113-1A. The transformants were selected in YPD plates containing G418 and the deletion of *URA3* was confirmed by their inability of growing in the absence of uracil. This way, when crossing the evolved strains (*MATa*) together with IMK440 (*MATα*) in a plate containing synthetic medium without uracil and with G418, only diploid cells are able to grow. The diploid cells were sporulated, their spores selected and plated on synthetic medium without uracil, selecting only the haploid segregants containing the native *URA3* locus [74]. The haploid segregants were screened for butanol tolerance and the one with the same tolerance as the evolved strain was selected. The selected haploid segregant was crossed with IMK439 or IMK440 (depending on its mating type) and a new sporulation and spore isolation was performed.

### Sequencing and analysis of the sequences

Genomic DNA of the strains CEN.PK113-7D, CEN.PK113-1A, IMS0344, IMS0346, IMS0348, IMS0350, IMS0351, IMS0353, IMS0355 and IMS0357 was prepared as described previously [40]. Libraries of 350-bp insert were constructed and paired end sequenced (100 base pair reads) using an illlumina HISeq 2000 sequencer (Baseclear BV, Leiden, The Netherlands). A minimum data quantity of 950 Mb was generated for each strain, representing a minimum 80-fold coverage. The sequence reads were mapped onto CEN.PK113-7D genome [43] using Burrows–Wheeler Alignment tool (BWA) and further processed using SAMtools [76-78]. Single-nucleotide variations were extracted from the mapping using SAMtools’ varFilter. Default settings were used, except that the maximum read depth was set to 400X (−D400). To minimize false positive mutation calls, custom Perl scripts were used for further mutation filtering: i) mutation calls containing ambiguous bases in mapping consensus were filtered out, ii) only the single-nucleotide variations with a quality of at least 20 were kept (variant quality is defined as the Phred-scaled probability that the mutation call is incorrect [79,80], iii) mutations with a. depth of coverage < 10X were discarded and iv) the mutations found in CEN.PK113-1A were subtracted from the list sequence Eventually, the single nucleotide variations were physically positioned and functionally annotated according to the CEN.PK113-7D sequence annotation [81]. The raw sequencing data of strains CEN.PK113-1A, IMS0344, IMS0346, IMS0348, IMS0350, IMS0351, IMS0353, IMS0355 and IMS0357 have been deposited as sequence read archives (SRA, http://www.ncbi.nlm.nih.gov/Traces/sra/sra.cgi?) under bioproject ID PRJNA191134.

### Fisher’s exact test

The 35 deletion strains exhibiting hyper sensitivity phenotype on 1% butanol were examined for enrichment in functional annotation in the GO [82] databases as previously described [83-85].

## Competing interests

The authors declare that they have no competing interest.

## Authors’ contributions

DGR, AJAvM, JTP and JMGD conceived and designed the experiments. Experimental work was carried out by DGR. Data analysis was performed by DGR, MvdB, AJAvM, JTP and JMGD. The manuscript was written by. DGR, AJAvM, JTP and JMGD. All authors approved the final version of the manuscript.

## Supplementary Material

Additional file 1: Table S1 Genes whose deletion results in *n*-butanol sensitivity in both BY4741 and CEN.PK113-7D genetic backgrounds. **Table S2**: Strains used in this study. **Table S3:** list of the primers used for the amplification of the deletion cassettes and the deletion confirmation of the 47 genes with a BSI value >10 in BY4741 deleted in CEN.PK113.7D). **Table S4**: Primers used for overexpressing genes whose deletions conferred higher butanol sensitivity in both BY4741 and CEN.PK113-7D [40]. **Table S5:** DNA cassettes used to reverse engineer the mutated alleles *rpn4-1* and *rtg1-1* in CEN.PK113-7D. **Table S6:** Primers used for the deletion of *URA3* and for the reverse engineering of the mutated alleles of *RPN4* and *RTG1* present in the evolved strains. Click here for file
