# Supplementary material for: Genome-scale analyses of butanol tolerance in Saccharomyces cerevisiae reveal an essential role of protein degradation
Source: Biotechnol Biofuels. 2013 Apr 3;6:48. doi: 10.1186/1754-6834-6-48 (PMC3621596; doi:10.1186/1754-6834-6-48)
Supplement: Additional file 1: Table S1 — Genes whose deletion results in n-butanol sensitivity in both BY4741 and CEN.PK113-7D genetic backgrounds. Table S2: Strains used in this study. Table S3: list of the primers used for the amplification of the deletion cassettes and the deletion confirmation of the 47 genes with a BSI value >10 in BY4741 deleted in CEN.PK113.7D). Table S4: Primers used for overexpressing genes whose deletions conferred higher butanol sensitivity in both BY4741 and CEN.PK113-7D [40]. Table S5: DNA cassettes used to reverse engineer the mutated alleles rpn4-1 and rtg1-1 in CEN.PK113-7D. Table S6: Primers used for the deletion of URA3 and for the reverse engineering of the mutated alleles of RPN4 and RTG1 present in the evolved strains. [file 1754-6834-6-48-S1.docx]

**Supplementary information.**

**Table S1:** genes whose deletion results in *n*-butanol sensitivity in both BY4741 and CEN.PK113-7D genetic backgrounds. The butanol Sensitivity index represents the ratio of the average OD660 of cultures grown without butanol over the average OD660 of cultures grown in presence of 1% butanol. The data presented are the average and standard deviation (sd) of at least 8 cultures. The genes shown in red have also been deleted in CEN.PK113-7D.

|  |  | **Without butanol** | | **1% butanol** | |
| --- | --- | --- | --- | --- | --- |
| **Gene name** | **BSI** | **Average OD_660_** | **sd** | **Average OD_660_** | **sd** |
| *SNT309* | 18.73 | 0.80 | 0.01 | 0.04 | 0.00 |
| *NKP2* | 18.34 | 0.80 | 0.02 | 0.04 | 0.00 |
| YKL118W | 18.28 | 0.76 | 0.01 | 0.04 | 0.00 |
| *SEC28* | 17.62 | 0.78 | 0.02 | 0.04 | 0.01 |
| *REG1* | 17.60 | 0.83 | 0.01 | 0.05 | 0.03 |
| *BRE5* | 17.18 | 0.78 | 0.02 | 0.05 | 0.00 |
| *VMA22* | 17.17 | 0.74 | 0.01 | 0.04 | 0.00 |
| YGL188C-A | 17.10 | 0.81 | 0.01 | 0.05 | 0.00 |
| *VMA7* | 16.69 | 0.75 | 0.02 | 0.04 | 0.00 |
| *EFG1* | 16.27 | 0.72 | 0.01 | 0.04 | 0.00 |
| *ALD6* | 16.21 | 0.70 | 0.01 | 0.04 | 0.00 |
| *GON7* | 15.91 | 0.67 | 0.02 | 0.04 | 0.00 |
| *UBP3* | 15.48 | 0.72 | 0.03 | 0.05 | 0.00 |
| YPL062W | 15.44 | 0.71 | 0.01 | 0.05 | 0.13 |
| YBL094C | 15.27 | 0.66 | 0.02 | 0.04 | 0.00 |
| *UAF30* | 15.27 | 0.75 | 0.01 | 0.05 | 0.00 |
| *GND1* | 14.96 | 0.62 | 0.01 | 0.04 | 0.00 |
| *BRO1* | 14.44 | 0.68 | 0.12 | 0.05 | 0.01 |
| *GPH1* | 14.24 | 0.80 | 0.02 | 0.06 | 0.01 |
| *UMP1* | 14.03 | 0.66 | 0.02 | 0.05 | 0.00 |
| *HTL1* | 13.95 | 0.60 | 0.04 | 0.04 | 0.01 |
| *VPS15* | 13.86 | 0.62 | 0.02 | 0.05 | 0.00 |
| YLR338W | 13.39 | 0.75 | 0.02 | 0.06 | 0.00 |
| *SHE4* | 13.30 | 0.62 | 0.02 | 0.05 | 0.00 |
| *SNF8* | 13.26 | 0.78 | 0.03 | 0.06 | 0.01 |
| *POL32* | 13.24 | 0.82 | 0.01 | 0.06 | 0.01 |
| *DHH1* | 13.08 | 0.67 | 0.02 | 0.05 | 0.00 |
| YLR224W | 12.85 | 0.79 | 0.01 | 0.06 | 0.14 |
| *ANP1* | 12.45 | 0.52 | 0.01 | 0.04 | 0.00 |
| *THP2* | 12.44 | 0.80 | 0.01 | 0.06 | 0.01 |
| *OCH1* | 12.21 | 0.61 | 0.01 | 0.05 | 0.01 |
| *MAP1* | 12.01 | 0.50 | 0.06 | 0.04 | 0.00 |
| *VRP1* | 11.86 | 0.71 | 0.01 | 0.06 | 0.01 |
| *ROX3* | 11.76 | 0.66 | 0.02 | 0.06 | 0.01 |
| *VPS34* | 11.75 | 0.52 | 0.06 | 0.04 | 0.00 |
| *GET1* | 11.63 | 0.79 | 0.02 | 0.07 | 0.01 |
| *SLA1* | 11.49 | 0.69 | 0.02 | 0.06 | 0.01 |
| *GEP5* | 11.12 | 0.67 | 0.01 | 0.06 | 0.03 |
| *PRE9* | 11.04 | 0.72 | 0.02 | 0.07 | 0.01 |
| *MFT1* | 11.02 | 0.78 | 0.01 | 0.07 | 0.05 |
| YDR157W | 11.02 | 0.51 | 0.07 | 0.05 | 0.03 |
| *HPR1* | 10.67 | 0.68 | 0.01 | 0.06 | 0.01 |
| *LTV1* | 10.66 | 0.76 | 0.02 | 0.07 | 0.01 |
| *GPD2* | 10.42 | 0.73 | 0.01 | 0.07 | 0.01 |
| *HOM2* | 10.28 | 0.81 | 0.04 | 0.08 | 0.03 |
| *GET2* | 10.26 | 0.79 | 0.01 | 0.08 | 0.16 |
| *PIH1* | 10.21 | 0.79 | 0.02 | 0.08 | 0.01 |
| *SWI6* | 10.18 | 0.68 | 0.02 | 0.07 | 0.01 |
| *MSE1* | 10.07 | 0.68 | 0.01 | 0.07 | 0.01 |
| *BUD20* | 10.03 | 0.73 | 0.01 | 0.07 | 0.04 |
| *SSQ1* | 9.91 | 0.57 | 0.02 | 0.06 | 0.00 |
| YKR023W | 9.75 | 0.56 | 0.01 | 0.06 | 0.01 |
| *CLC1* | 9.70 | 0.72 | 0.02 | 0.07 | 0.01 |
| *HIR1* | 9.52 | 0.75 | 0.02 | 0.08 | 0.10 |
| *YHR175W-A* | 9.08 | 0.61 | 0.02 | 0.07 | 0.01 |
| *RRG7* | 8.39 | 0.70 | 0.01 | 0.08 | 0.01 |
| *ATP15* | 8.00 | 0.61 | 0.01 | 0.08 | 0.01 |
| *MDM34* | 7.38 | 0.75 | 0.01 | 0.10 | 0.02 |
| *RPS24A* | 7.17 | 0.78 | 0.02 | 0.11 | 0.04 |
| *SPT20* | 6.83 | 0.64 | 0.02 | 0.09 | 0.02 |
| *MDM12* | 6.77 | 0.76 | 0.02 | 0.11 | 0.02 |
| *GPH1* | 6.75 | 0.71 | 0.01 | 0.10 | 0.02 |
| *RPL41B* | 5.89 | 0.78 | 0.02 | 0.13 | 0.03 |
| *SER1* | 5.66 | 0.77 | 0.02 | 0.14 | 0.06 |
| *VPS65* | 5.65 | 0.66 | 0.03 | 0.12 | 0.02 |
| *HFI1* | 5.40 | 0.69 | 0.03 | 0.13 | 0.16 |
| *DID4* | 5.32 | 0.71 | 0.01 | 0.13 | 0.03 |
| *SRB5* | 5.15 | 0.74 | 0.02 | 0.14 | 0.02 |
| *FMP37* | 5.12 | 0.75 | 0.02 | 0.15 | 0.04 |
| *LYS4* | 5.00 | 0.71 | 0.02 | 0.14 | 0.01 |
| *SPF1* | 4.97 | 0.74 | 0.02 | 0.15 | 0.02 |
| *ATP12* | 4.78 | 0.27 | 0.01 | 0.06 | 0.00 |
| *AGP2* | 4.70 | 0.70 | 0.02 | 0.15 | 0.04 |
| *FCY22* | 4.63 | 0.66 | 0.05 | 0.14 | 0.01 |
| *RPS8A* | 4.49 | 0.83 | 0.01 | 0.19 | 0.04 |
| *AEP3* | 4.36 | 0.75 | 0.01 | 0.17 | 0.03 |
| *CCR4* | 4.14 | 0.76 | 0.01 | 0.18 | 0.07 |
| *STP22* | 4.08 | 0.78 | 0.02 | 0.19 | 0.04 |
| YNL198C | 3.93 | 0.74 | 0.01 | 0.19 | 0.03 |
| *YKE2* | 3.93 | 0.77 | 0.01 | 0.20 | 0.01 |
| *MEH1* | 3.90 | 0.78 | 0.02 | 0.20 | 0.03 |
| *GCR2* | 3.74 | 0.74 | 0.02 | 0.20 | 0.04 |
| *HOF1* | 3.56 | 0.72 | 0.01 | 0.20 | 0.09 |
| *PRO2* | 3.51 | 0.41 | 0.05 | 0.12 | 0.03 |
| *PIM1* | 3.47 | 0.75 | 0.01 | 0.22 | 0.05 |
| *MGM1* | 3.37 | 0.72 | 0.01 | 0.21 | 0.09 |
| *TRP3* | 3.35 | 0.72 | 0.01 | 0.21 | 0.02 |
| *SWI4* | 3.32 | 0.70 | 0.02 | 0.21 | 0.04 |
| *TDA9* | 3.24 | 0.74 | 0.01 | 0.23 | 0.12 |
| YGL042C | 3.24 | 0.81 | 0.01 | 0.25 | 0.03 |
| *FUM1* | 3.05 | 0.75 | 0.01 | 0.25 | 0.04 |
| *FYV5* | 2.93 | 0.73 | 0.02 | 0.25 | 0.06 |
| *OPI10* | 2.88 | 0.83 | 0.01 | 0.29 | 0.08 |
| YGL072C | 2.87 | 0.74 | 0.02 | 0.26 | 0.04 |
| *CDC10* | 2.81 | 0.75 | 0.02 | 0.27 | 0.06 |
| *SLS1* | 2.77 | 0.68 | 0.01 | 0.24 | 0.07 |
| *POC4* | 2.75 | 0.75 | 0.02 | 0.27 | 0.05 |
| *MRPL36* | 2.73 | 0.67 | 0.01 | 0.25 | 0.05 |
| YMR326C | 2.66 | 0.73 | 0.02 | 0.28 | 0.06 |
| YNL171C | 2.56 | 0.79 | 0.01 | 0.31 | 0.03 |
| *RRG9* | 2.50 | 0.75 | 0.01 | 0.30 | 0.05 |
| *YAF9* | 2.49 | 0.79 | 0.02 | 0.32 | 0.06 |
| *ADE5,7* | 2.46 | 0.80 | 0.01 | 0.33 | 0.05 |
| *RTG1* | 2.43 | 0.51 | 0.01 | 0.21 | 0.05 |
| *CTF4* | 2.31 | 0.73 | 0.02 | 0.31 | 0.05 |

**Table S2**: Strains used in this study.

| **Strain name** | **Description** | **Source** |
| --- | --- | --- |
| BY4741 | MATa his3Δ1 leu2Δ met15Δ ura3Δ | Euroscarf, |
| Yeast KO collection | MATa his3Δ1 leu2Δ met15Δ ura3Δ *ORFΔ*::KanMX | OpenBiosystems |
| CEN.PK113-7D | *MATa* | Euroscarf |
| CEN.PK113-1A | *MATα* | Euroscarf |
| IMK439 | *MATα ura3Δ*::KanMX | This study |
| IMK440 | *MATa* *ura3Δ*::KanMX | This study |
| IMK356 | *MATa pre9*Δ::KanMX | This study |
| IMK357 | *MATa* YLR224wΔ::KanMX | This study |
| IMK358 | *MATa bre5*Δ::KanMX | This study |
| IMK359 | *MATa ubp3*Δ::KanMX | This study |
| IMK360 | *MATa ump1*Δ::KanMX | This study |
| IMK361 | *MATa pih1*Δ::KanMX | This study |
| IMK362 | *MATa get2*Δ::KanMX | This study |
| IMK379 | *MATa stp22*Δ::KanMX | This study |
| IMK380 | *MATa did4*Δ::KanMX | This study |
| IMK365 | *MATa snf8*Δ::KanMX | This study |
| IMK366 | *MATa bro1*Δ::KanMX | This study |
| IMK367 | *MATa vma7*Δ::KanMX | This study |
| IMK368 | *MATa get1*Δ::KanMX | This study |
| IMK370 | *MATa ssq1*Δ::KanMX | This study |
| IMK371 | *MATa vps34*Δ::KanMX | This study |
| IMK372 | *MATa vma22*Δ::KanMX | This study |
| IMK403 | *MATa bud20*Δ::KanMX | This study |
| IMK390-DG | *MATa gep5*Δ::KanMX | This study |
| IMK391-DG | *MATa mtf1*Δ::KanMX | This study |
| IMK363 | *MATa she4*Δ::KanMX | This study |
| IMK364 | *MATa mse1*Δ::KanMX | This study |
| IMK395-DG | *MATa gdp2*Δ::KanMX | This study |
| IMK414 | *MATa tph2*Δ::KanMX | This study |
| IMK404 | *MATa gnd1*Δ::KanMX | This study |
| IMK430 | *MATa swi6*Δ::KanMX | This study |
| IMK405 | *MATa ltv1*Δ::KanMX | This study |
| IMK392-DG | *MATa nkp2*Δ::KanMX | This study |
| IMK397-DG | *MATa hom2*Δ::KanMX | This study |
| IMK398-DG | *MATa ald6*Δ::KanMX | This study |
| IMK399-DG | *MATa gph1*Δ::KanMX | This study |
| IMK394-DG | *MATa sla1*Δ::KanMX | This study |
| IMK400 | *MATa sec28*Δ::KanMX | This study |
| IMK410 | *MATa snt309*Δ::KanMX | This study |
| IMK407 | *MATa reg1*Δ::KanMX | This study |
| IMK424 | *MATa htl1*Δ::KanMX | This study |
| IMK408 | *MATa pol32*Δ::KanMX | This study |
| IMK415 | *MATa dhh1*Δ::KanMX | This study |
| IMK416 | *MATa vrp1*Δ::KanMX | This study |
| IMK417 | *MATa vps15*Δ::KanMX | This study |
| IMK418 | *MATa map1*Δ::KanMX | This study |
| IMK401 | *MATa rox3*Δ::KanMX | This study |
| IMK402 | *MATa anp1*Δ::KanMX | This study |
| IMK425 | *MATa efg1*Δ::KanMX | This study |
| IMK420 | *MATa* YGL188c-AΔ::KanMX | This study |
| IMI176 | *MATa PRE9p*::KanMX-*TPI1p* | This study |
| IMI088 | *MATa* YLR224w*p*::KanMX-*TPI1p* | This study |
| IMI089 | *MATa BRE5p*::KanMX-*TPI1p* | This study |
| IMI174 | *MATa UBP3p*::KanMX-*TPI1p* | This study |
| IMI177 | *MATa UMP1p*::KanMX-*TPI1p* | This study |
| IMI151 | *MATa PIH1p*::KanMX-*TPI1p* | This study |
| IMI152 | *MATa GET2p*::KanMX-*TPI1p* | This study |
| IMI153 | *MATa STP22p*::KanMX-*TPI1p* | This study |
| IMI154 | *MATa DID4p*::KanMX-*TPI1p* | This study |
| IMI155 | *MATa SNF8p*::KanMX-*TPI1p* | This study |
| IMI187 | *MATa BRO1p*::KanMX-*TPI1p* | This study |
| IMI156 | *MATa VMA7p*::KanMX-*TPI1p* | This study |
| IMI157 | *MATa GET1p*::KanMX-*TPI1p* | This study |
| IMI159 | *MATa VPS34p*::KanMX-*TPI1p* | This study |
| IMI161 | *MATa VMA22p*::KanMX-*TPI1p* | This study |
| IMI163 | *MATa GEP5p*::KanMX-*TPI1p* | This study |
| IMI164 | *MATa MTF1p*::KanMX-*TPI1p* | This study |
| IMI165 | *MATa SHE4p*::KanMX-*TPI1p* | This study |
| IMI167 | *MATa MSE1p*::KanMX-*TPI1p* | This study |
| IMI168 | *MATa THP2p*::KanMX-*TPI1p* | This study |
| IMI117 | *MATa GND1p*::KanMX-*TPI1p* | This study |
| IMI169 | *MATa SWI6p*::KanMX-*TPI1p* | This study |
| IMI136 | *MATa NKP2p*::KanMX-*TPI1p* | This study |
| IMI146 | *MATa HOM2p*::KanMX-*TPI1p* | This study |
| IMI121 | *MATa ALD6p*::KanMX-*TPI1p* | This study |
| IMI124 | *MATa SLA1p*::KanMX-*TPI1p* | This study |
| IMI137 | *MATa SEC28p*::KanMX-*TPI1p* | This study |
| IMI125 | *MATa SNT309p*::KanMX-*TPI1p* | This study |
| IMI126 | *MATa REG1p*::KanMX-*TPI1p* | This study |
| IMI143 | *MATa HTL1p*::KanMX-*TPI1p* | This study |
| IMI144 | *MATa POL32p*::KanMX-*TPI1p* | This study |
| IMI141 | *MATa DHH1p*::KanMX-*TPI1p* | This study |
| IMI142 | *MATa VRP1p*::KanMX-*TPI1p* | This study |
| IMI178 | *MATa VPS15p*::KanMX-*TPI1p* | This study |
| IMI148 | *MATa ANP1p*::KanMX-*TPI1p* | This study |
| IMS0344 | *MATa rpn4-1 rtg1-1 ubr1-1 ssk2-1* | This study |
| IMS0345 | *MATa*/*MATα* *RPN4/rpn4-1 RTG1/rtg1-1 UBR1/ubr1-1 SSK2/ssk2-1 URA3/ura3Δ*::KanMX | This study |
| IMS0346 | *MATα* *rpn4-1 rtg1-1 ubr1-1 ssk2-1* | This study |
| IMS0347 | *MATa*/*MATα* *RPN4/rpn4-1 RTG1/rtg1-1 UBR1/ubr1-1 SSK2/ssk2-1 URA3/ura3Δ*::KanMX | This study |
| IMS0348 | *MATα* *rpn4-1 rtg1-1 ubr1-1 ssk2-1* | This study |
| IMS0349 | *MATa*/*MATα* *RPN4/rpn4-1 RTG1/rtg1-1 UBR1/ubr1-1 SSK2/ssk2-1 URA3/ura3Δ*::KanMX | This study |
| IMS0350 | *MATa* *rpn4-1 rtg1-1 ssk2-1* | This study |
| IMS0351 | *MATa rpn4-2 rtg1-2 ubr1-2 nma111-2 rpl10-2 sto1-2 sst4-2* | This study |
| IMS0352 | *MATa*/*MATα* *RPN4/rpn4-2 RTG1/rtg1-2 UBR1/ubr1-2 NMA111/nma111-2 RPL10/rpl10-2 STO1/sto1-2 SST4/sst4-2 URA3/ura3Δ*::KanMX | This study |
| IMS0353 | *MATa* *rpn4-2 rtg1-2 nma111-2 rpl10-2* | This study |
| IMS0354 | *MATa*/*MATα* *RPN4/rpn4-1 RTG1/rtg1-2 NMA111/nma111-2* *RPL10/rpl10-2 URA3/ura3Δ*::KanMX | This study |
| IMS0355 | *MATa* *rpn4-2 rtg1-2 nma111-2 rpl10-2* | This study |
| IMS0356 | *MATa*/*MATα RPN4/rpn4-2 RTG1/rtg1-2 NMA111/nma111-2* *RPL10/rpl10-2 URA3/ura3Δ*::KanMX | This study |
| IMS0357 | *MATa* *rpn4-2 rtg1-2 nma111-2* | This study |
| IMI218 | *MATa RPN4p*::KanMX *RPN4p-rpn4-1* | This study |
| IMI238 | *MATa RTG1p*::KanMX *RTG1p-rtg1-1* | This study |

**Table S3:** list of the primers used for the amplification of the deletion cassettes and the deletion confirmation of the 47 genes with a BSI value >10 in BY4741 deleted in CEN.PK113.7D). The deletion cassettes containing the KanMX marker were amplified from the gDNA of the strain in the collection deleted in the corresponding gene (Table S1). The primers used to amplify the deletion cassette were systematically named (–DF and –DR, stating for deletion forward and deletion reverse, respectively). The confirmation of the deletion was done by PCR amplification using the gDNA of the transformants as a template, with the primers named with the suffixes –DCF (deletion confirmation forward) and KanMX-DCR (deletion confirmation reverse). Alternatively we used the primers KanMX-DCF (deletion confirmation forward) and the primers named with the suffix -DCR (deletion confirmation reverse).

| **Target gene** | **Primer name** | **Sequence** |
| --- | --- | --- |
| KanMX (marker) | KanMX-DCF | ATACTAACGCCGCCATCC |
|  | KanMX-DCR | GACGAGGCAAGCTAAACAG |
| *PRE9* | YGR135w-DF | GCAGAGCGAAGAGAACAGAC |
|  | YGR135w-DR | CTCGAGCGATTCCGATCTTG |
|  | YGR135w-DCF | AGCTCGATGTTGGAGACAG |
| YLR224w | YLR224w-DF | GTTTCCGCTGTTTGCTTG |
|  | YLR224w-DR | GTAGGTAGGTGCATAGAC |
|  | YLR224w-DCF | AGGCCATCTCAGACAAGTAG |
| *BRE5* | YNR051c-DF | CGGCGGACGTTAAACTAAG |
|  | YNR051c-DR | CTTCCGCGCCAGTATATTAG |
|  | YNR051c-DCR | TTTCGCGCATCTACTCTACG |
| *UBP3* | YER151c-DF | AGCGAAAGGGAAGAGAAGTG |
|  | YER151c-DR | CTGCTTAACACGCAGAACG |
|  | YER151c-DCF | GGGTCAGTCATGCTTTAGG |
| *UMP1* | YBR173c-DF | TCGGGTAACCATGCAGTG |
|  | YBR173c-DR | ATGTGGGCTGAGAAGTTGAG |
|  | YBR173c-DCF | ATCTACCGCCAGTGATGC |
| *PIH1* | YHR034c-DF | CACATAGGCACTTCCTTGG |
|  | YHR034c-DR | TTTGGGCTACAGTGTGAGTG |
|  | YHR034c-DCR | TCAAGGGCTTACCCAGTG |
| *GET2* | YER083c-DF | GGAGGGAGGGAGAAGTTTG |
|  | YER083c-DR | CCCATTGTGTCGTTGGAATC |
|  | YER083c-DCF | GATGGCTACTTGGGTTGAG |
| *STP22* | YCL008c-DF | ACGGCACTCCACAACTAC |
|  | YCL008c-DR | CCGCTACATAGTCCACATCC |
|  | YCL008c-DCF | CGATTCTTTAGCGCTGCTG |
| *DID4* | YKL002w-DF | TCAGAGGGAGCTTGAAAGAG |
|  | YKL002w-DR | GACCAGAAGACGGTTGAAG |
|  | YKL002w-DCF | TTCACCCTTGGACACAGAAC |
| *SNF8* | YPL002c-DF | TTTGAGGCGATGCAGTAG |
|  | YPL002c-DR | CCCTGGTTTGCGTTGATG |
|  | YPL002c-DCF | TTGGCGCTTGAAGTCCAC |
| *BRO1* | YPL084w-DF | GCAAGCAAGGCTTCAGTTAC |
|  | YPL084w-DR | GTACCCACCTCACATACAG |
|  | YPL084w-DCF | AAACAGCGAGAGGCTATG |
| *VMA7* | YGR020c-DF | TACGCCGGTAAAGGGAAG |
|  | YGR020c-DR | GGCGGGTATTCACAATAGG |
|  | YGR020c-DCF | GCAGTTCACCCAGATACC |
| *GET1* | YGL020c-DF | TTGCACGTACCAACTACCTC |
|  | YGL020c-DR | TGGAGACGGAGGACATTAAG |
|  | YGL020c-DCR | GCGGATCTTGTTGAACATGG |
| *SSQ1* | YLR369w-DF | GAGCGGCCTTTCCTTAAC |
|  | YLR369w-DR | TTGGTTGGCGTTCACAAG |
|  | YLR369w-DCF | TGTTCCACGAACTGACTG |
| *VPS34* | YLR240w-DF | AGATCCGGCATCAAACAC |
|  | YLR240w-DR | GCCGACTTGAGCCTTTC |
|  | YLR240w-DCF | TGGCGAATACACCTCGAC |
| *VMA22* | YHR060w-DF | GACGCTTTCGCAAGATCTC |
|  | YHR060w-DR | CTACATACGCCCGAATTAGG |
|  | YHR060w-DCF | TTGGTGGTGAGAGGAAGAG |
| *BUD20* | BG18-DF | TGGAAAGTAGCGGGAGATG |
|  | BG18-DR | AGCGATTCGAGTTGACTG |
|  | BG18-DCF | CCACGGAAATAACCGCTAC |
| *GEP5* | BG19-DF | AGTCTTTCGCACCTTCAG |
|  | BG19-DR | CCGACGTTGCTAGTTTCTG |
|  | BG19-DCF | CAGCGCATCAGAAAGCAAG |
| *MFT1* | BG20-DF | TGGTGAACCTCCTTCGATAG |
|  | BG20-DR | GAAGGTGACGCACCATAC |
|  | BG20-DCF | GAACCTCGTGGTCAGGATAC |
| *SHE4* | BG21-DF | GGCACGAAGGCTTAAATCC |
|  | BG21-DR | GCTGCACGAAGTCGTAAC |
|  | BG21-DCF | TACTCTGGCGCAGTAGTTC |
| *UAF30* | BG22-DF | GCAGTAGTCCAAGATGAG |
|  | BG22-DR | CACCCGGGAATGTTAACG |
|  | BG22-DCF | CCCAATCGATCCGCTAACC |
|  | BG22-DCFII | CGGTGAATGGGTTCCAAAG |
| *MSE1* | BG23-DF | AATTCGAGGCACCAAAGC |
|  | BG23-DR | CGGTTTCGTTCGAGAAAGG |
|  | BG23-DCF | CTAGTGTCAGCTGTCTTACC |
| *GPD2* | BG24-DF | AGCAGCTCTTCTCTACCC |
|  | BG24-DR | AGTGTACAGGGTGTCGTATC |
|  | BG24-DCF | ACGGACCTATTGCCATTG |
| *THP2* | BG25-DF | GCTGCAGAAGTGCAGTATC |
|  | BG25-DR | CCGAGGACTGATTCTTGATG |
|  | BG25-DCF | AGTACCGCAGTACTTGGTC |
| *GND1* | BG26-DF | GCTGCAGTATTGTTCCTGAG |
|  | BG26-DR | CCTGTTTGCCTTTCCTTACG |
|  | BG26-DCF | AAATGGGCCTGATGTTCG |
| *SWI6* | BG27-DF | GTCTTCATCTGGACCGTTTG |
|  | BG27-DR | GCAAACCATTGGACGAAGAG |
|  | BG27-DCF | CCTTGCAGCGTTACTAGC |
| *LTV1* | BG28-DF | TCAGGGTTCTTACCGAATCC |
|  | BG28-DR | CGGCTCTTTGAAGCAATG |
|  | BG28-DCF | GCGTCTATTTCTGCACATGG |
| *NKP2* | BG30-DF | CTTGTTACGCCACGAACAG |
|  | BG30-DR | ACGGGTACTGCATGAACG |
|  | BG30-DCF | TTGCGGTGCCTTTCC |
| *HOM2* | BG32-DF | AGCGGCCTCAAAGTACC |
|  | BG32-DR | TTTCTTGGGTCAGCGAGAG |
|  | BG32-DCF | CCATTCACATACGCCTTCTG |
| *ALD6* | BG34-DF | GCCTGGCGTGTTTAACAAG |
|  | BG34-DR | AGGCACAAGCCTGTTCTC |
|  | BG34-DCF | GACCATGTGGGCAAATTCG |
| *GPH1* | BG35-DF | GCCTTCCCGTTTCTTGTTG |
|  | BG35-DR | ATAAGGACCGCCTTACCC |
|  | BG35-DCF | TAACAAGGAACGCCTTCCC |
| *SLA1* | BG37-DF | CCTTCCACTCATGCGAAATC |
|  | BG37-DR | GTCGACTCCACCATTTCAAC |
|  | BG37-DCF | TAGAGAACATGCCTGCGTAG |
| *SEC28* | BG38-DF | CTCCAACGCAACGATCAG |
|  | BG38-DR | TCTGCCAGGATTAGCTAC |
|  | BG38-DCF | CCACAAGCTGGTGAAATGG |
| *SNT309* | BG39-DF | CCCTGCAAATCACACTAGTC |
|  | BG39-DR | GCGCTAAGGACTTCTATGG |
|  | BG39-DCF | GGGTATATGGTCGCCTCTG |
| *REG1* | BG40-DF | ACCACCTCCTGAAAGAGAAC |
|  | BG40-DR | GCCAGTCGATTACAGCTTAC |
|  | BG40-DCF | GGAGACGCGATTTCAAACG |
| *HTL1* | BG41-DF | ATCCGCCACATAAGATGC |
|  | BG41-DR | CTGGATACATTCGCGGTAAC |
|  | BG41-DCF | GATGTCCATGACGCTTTG |
| *POL32* | BG42-DF | TCCGACGGAAGTAGTAAACG |
|  | BG42-DR | GTGGCGACAGTCATTGAAG |
|  | BG42-DCF | TGTCCTCGGATCGAAACC |
| *DHH1* | BG43-DF | CATAACCGCATCGCCATTC |
|  | BG43-DR | AAACGGTGCGCAAATGAG |
|  | BG43-DCF | GGGCGATTGTAACATTGGG |
| *VRP1* | BG44-DF | ACCTAGTCACTGCTTACG |
|  | BG44-DR | CAGCTGTGGACTGGCTATC |
|  | BG44-DCF | CGGTTTGTCCGCTACATTG |
| *OCH1* | BG45-DF | TCTCGCCAATCCACATTCTC |
|  | BG45-DR | ACGGAAGGACGTTGAGATAG |
|  | BG45-DCF | GCTGGGCCTCAACTAAAC |
| *VPS15* | BG46-DF | TCCACGAGACAGTACCATTG |
|  | BG46-DR | TCTTTGCGGTGATGATGG |
|  | BG46-DCF | AGGACGGTAGAAAGACCATC |
| *MAP1* | BG48-DF | TTGCGGCGTTTGCAG |
|  | BG48-DR | CAATTCGGACTCGGGAAAG |
|  | BG48-DCF | GTGGCGATTCTGAGGAC |
| *GON7* | BG49-DF | TTACGGCCTTGTCAGGTC |
|  | BG49-DR | CAAACCGGCAGGATGATTC |
|  | BG49-DCF | CGCGAATGCTCTAGTGATG |
|  | BG49-DFII | TGTCTTAGCACCACAGAG |
|  | BG49-DRII | GCCCTTCGTTCTTGGTTTG |
|  | BG49-DCFII | TACAGCCGCACGTAGATCG |
| *ROX3* | BG50-DF | GAGCCTCTTCATTCCTTTCC |
|  | BG50-DR | GCACAAACGTGCCACTTC |
|  | BG50-DCF | TCGACCCGTTGTTCCTG |
| *ANP1* | BG51-DF | CGAGTGTTGGTGCGATTTAC |
|  | BG51-DR | CGAGCCGTGTATAGTGATTG |
|  | BG51-DCF | TGAACCGGGCTCATCTG |
| *EFG1* | BG52-DF | ACCATCTACAGACCCAAAGG |
|  | BG52-DR | TCGTGGCGAAGAATTGC |
|  | BG52-DCF | AAGAATCTGGCGCAGATG |
|  | BG52-DCFII | AAGGCTCTTGGGTCATCTC |
| YGL188c-A | BG53-DF | CACCGCTTCGTTCTCATTG |
|  | BG53-DR | CGTGACCTCTACCTTTCTTG |
|  | BG53-DCF | GCGGAACTCGAAGTGTC |

**Table S4**: Primers used for overexpressing genes whose deletions conferred higher butanol sensitivity in both BY4741 and CEN.PK113-7D. The promoter replacement cassettes containing the KanMX marker and the promoter from *TPI1* were amplified from the plasmid pUG6-TPI1 prom [1]. The primers used for the amplification have been systematically named as followed: gene name–OF or –OR, for overexpression forward and overexpression reverse, respectively. The confirmation of the correct promoter replacement was done by PCR amplification using the gDNA of the transformants as a template, with the primers named with the suffixes –OCR (overexpression confirmation reverse) and TPIprom-ICF (insertion confirmation forward).

| **Target gene** | **Primer name** | **Sequence** |
| --- | --- | --- |
| TPI1  promoter | TPI1prom-ICF | CGCATGCTAATGCAAAGG |
| *PRE9* | BG1-OFII | TGGATATCTATGTAATAAGGAAACATTGGCAGAGCGAAGAGAACAGACTGCCGGATATGGACAATCATCGACAGCTGAAGCTTCGTACGC |
|  | YGR135w-OR | TCCCTCAGGGGAGAAAATTGTTGTCCTGGAATCGTATCTTCTGGAACCCATCACTATAGGGAGACCGGCAG |
|  | YGR135w-OCR | ATCCCAATTGCGGTACCTG |
| YLR224w | YLR224w-OF | GAATATTACTATGTTTGAAATGGTTATTGCTTATGATGCTCACCGAAGTTAAACAGTTTAGCAACGTGCCCCAGCTGAAGCTTCGTACGC |
|  | YLR224w-OR | TAAATGTATCTCCAGTGGTAAATCCATCAAGCTGCTATCGCTCTGATTCATCACTATAGGGAGACCGGCAG |
|  | YLR224w-OCR | TCCACGCCAAACTCTTCTC |
| *BRE5* | YNR051c-OF | CAATAAGCACTCTTCCACAGGAGATTGATGCGTTATTCCCGTATAGTTATCGAAACCATTCGTCTCAATCGCAGCTGAAGCTTCGTACGC |
|  | YNR051c-OR | GTAGTAGTTTTGCAAAAAGGCAAAACATATGTCTTGAACGGTAACACCCATCACTATAGGGAGACCGGCAG |
|  | YNR051c-OCR | CCGTTGGAAGAACGTCATC |
| *UBP3* | BG4-OFII | CAAGCAGCGAAAAAGCGAAAGGGAAGAGAAGTGTTTCTAGAGAAGAAAGTCTTCATGTCGGCAGTTGCGCTCAGCTGAAGCTTCGTACGC |
|  | YER151c-OR | TTTCGGGTACATCGAGTACGACTCTTCTTTGTTAGCGTCTTGCATGTTCATCACTATAGGGAGACCGGCAG |
|  | YER151c-OCR | GCCGTACATCTGCAAAGG |
| *UMP1* | BG5-OFII | CACTCCTCGGGTAACCATGCAGTGGCAATTTTATCCTACCTGAGGTTATTAAGCGCGGCGTCATCTTTAGTCAGCTGAAGCTTCGTACGC |
|  | YBR173c-OR | TTGATCTGTAGAGACTTGAGATTTAAAGGTATCTTGTGGGACGATATTCATCACTATAGGGAGACCGGCAG |
|  | YBR173c-OCR | TACTGCACCGCCTTCTTG |
| *PIH1* | BG6-OFII | GAAGACAATAATGATAGAAAAAAACTAGGAATTCTTGAATTCATATGCATGCTCAGTCAGTAGGAGTAATGCAGCTGAAGCTTCGTACGC |
|  | YHR034c-OR | GTCCTCGTTTCTATGACGTTGCTTAATTGGTCTCAATAAGAAATCGGCCATCACTATAGGGAGACCGGCAG |
|  | YHR034c-OCR | GGCCCATTAGCGGATAATAG |
| *GET2* | BG7-OFII | GATGGCTACTTGGGTTGAGCATTACTCTCTTTGTGAAAAGGGAGGGAGGGATATAGTCACGCAGCACGACGCAGCTGAAGCTTCGTACGC |
|  | YER083c-OR | TCTCCTTTCCCTTAGTAACCTGCGCTTTTCCGCCTCTGTTAATTCAGACATCACTATAGGGAGACCGGCAG |
|  | YER083c-OCR | CGCTAGGAGCGTCTAAAGG |
| *STP22* | BG8-OFII | CACAGAGCGAGAATAAAAAGTTACGGCAAAATTTTGTGGGCAGTTGGTATCCCAGTCTTCAAACGTGTTCACAGCTGAAGCTTCGTACGC |
|  | YCL008c-OR | CAGCCAATTGACCACCGCCTCGGGAACAGAGATCTTGCCGTTTGCAGACATCACTATAGGGAGACCGGCAG |
|  | YCL008c-OCR | CCAGCAGCGCTAAAGAATC |
| *DID4* | BG9-OFII | TGTATTTGGATCAGTTATTTTAGTGAACATACTAACGTTAATTATTTGAGTGAGAGCACCGAACACTCGTACAGCTGAAGCTTCGTACGC |
|  | YKL002w-OR | ACTTCGAACGGCCTGTATTCTCAATGATATCGCCTGAAGTTGAGCTTTCATCACTATAGGGAGACCGGCAG |
|  | YKL002w-OCR | TGGCCTCGCTCATAGAAC |
| *SNF8* | BG10-OFII | GGTGTGCTATTTCAAGAACTTAATCCATCAATTCTGGGATATATTATAACCTATAGTCCAGTCCAGGCACGCAGCTGAAGCTTCGTACGC |
|  | YPL002c-OR | GTACTTTCCATCCTTCAACTCGTCAAAGGCTGCCAGTCCAAACTGTTTCATCACTATAGGGAGACCGGCAG |
|  | YPL002c-OCR | CCAGTCGTTCTTGGAATACC |
| *BRO1* | BG11-OFII | CCTCATTTTCTTGCTTCGATTAATGCAGGACGCCGGTTCCGGGTTAGGATCTACAGCAGGATAACCTCGGCCAGCTGAAGCTTCGTACGC |
|  | YPL084w-OR | ATCCAGCTTCTCTGTGTCTTTTAGCTTTAGGTCAAATAAGTAAGGTTTCATCACTATAGGGAGACCGGCAG |
|  | YPL084w-OCR | GGTGCTAGTTCTCCATTTGC |
| *VMA7* | BG12-OFII | GGCGAACGATAACAGGAAAACCAACGTGAATTGCAAGCACTACATTTATTAACAGGTCCGTAACTTGGTAGCAGCTGAAGCTTCGTACGC |
|  | YGR020c-OR | TGTAGTATCTTCGTCAGCTATCACAGCTATAAGAGTACGTTTCTCAGCCATCACTATAGGGAGACCGGCAG |
|  | YGR020c-OCR | TCCGCGATATGTTGGTTG |
| *GET1* | BG13-OFII | TTAGACACTTACCAGTTGATGTTTTCACTTTTTCTTTCCTTCCCAAGAGTGCCGGTTTGAGAATATCTTCCCAGCTGAAGCTTCGTACGC |
|  | YGL020c-OR | AAACTTTGTGACCACAATAAAGAATATCGCTACCGCTGCTGCCCAATGCATCACTATAGGGAGACCGGCAG |
|  | YGL020c-OCR | CGCGGAGATAGAGTTGTTG |
| *VPS34* | BG15-OFII | GTCCTTCATTCGGTATATGCAGATCCGGCATCAAACACTTTTTCAAGAAAGCGAACTGAAGGCATATTTCCCAGCTGAAGCTTCGTACGC |
|  | YLR240w-OR | GGGAACATCCAGATCCTGTGAGACACAGAATGTTATGTTGTTCAGTGACATCACTATAGGGAGACCGGCAG |
|  | YLR240w-OCR | CGGCGTGTATATCGGAAG |
| *VMA22* | BG17-OFII | GTTGTCCTTCATTCATGTTGATATTTATATGAAACGCTGACTGGGCCTTTCACATGGTTGAAAGCGCGTCACAGCTGAAGCTTCGTACGC |
|  | YHR060w-OR | GTACTGTTCATCTGTAGTATCCATGTTCTGTGCCATCCTTGTTTCACTCATCACTATAGGGAGACCGGCAG |
|  | YHR060w-OCR | CTTCTGCAACTGCTCTAAGG |
| *GEP5* | BG19-OFII | ACCTTCAGTAGAAAAATAGGAAGAATCCCCATCGCGGCGGACATAAAGCATTGAGGCCAACAAGACGGCACAGCTGAAGCTTCGTACGC |
|  | BG19-OR | GGCGTAGACTCTATGACCGGCAGTAATAAGGCGTTAACCTGGGACGCCATCACTATAGGGAGACCGGCAG |
|  | BG19-OCR | GTGAGTGTGGTCGTCAATG |
| *MFT1* | BG20-OFII | TAGAATACAAACTGCTATAATAATCAAGGCCGAGATACATAGTTCCGCGGCCTGTGTATAGATTGAGGCTCAGCTGAAGCTTCGTACGC |
|  | BG20-OR | TAGTGCACTTTGGTTCTAACTTGGTCTATTTGTTTTTGTGACAGAGGCATCACTATAGGGAGACCGGCAG |
|  | BG20-OCR | CTCCTGTTGGCATGTTTCC |
| *SHE4* | BG21-OFII | GCACGAAGGCTTAAATCCAATTACGAATCTTCTACTTAATAGTGAAGACGTGCACGAGTCTCCAGTGTTGCAGCTGAAGCTTCGTACGC |
|  | BG21-OR | TCAATAGTAGAGCTATCGATTGGATCATTCCCTTTCTCACACAGTGGCATCACTATAGGGAGACCGGCAG |
|  | BG21-OCR | TACGCGGGACTCAGAATG |
| *MSE1* | BG23-OFII | TGCAATAATTAATTTCTTATATTAAATTCGAGGCACCAAAGCAAGCAACCATGTCATGCTGCACTTCCGTCAGCTGAAGCTTCGTACGC |
|  | BG23-OR | AGCTTCGAAGGTGAACAATAAGACCGTGTCGGTATTCTCAACATGATCATCACTATAGGGAGACCGGCAG |
|  | BG23-OCR | GAAGGCGCAAACCTAGTTC |
| *THP2* | BG25-OFII | ATGTGAGTACCGCAGTACTTGGTCTGCTATTATGAAGTGTTTGTTTTACCTTTAGGCGTAGCACTCGCTGCAGCTGAAGCTTCGTACGC |
|  | BG25-OR | TCCTCCTCGCAGAGAGATTCAAAATACGTACGACCTTCTTCCTTTGTCATCACTATAGGGAGACCGGCAG |
|  | BG25-OCR | TCTAGGGTCTGCCAATACTG |
| *GND1* | BG26-OF | ACCTGCTACCTCTCTGTTCTTTCGTCCCGCCTCGAGGTTGTGCTGCTTTTTTTATCCTGGCACGCTGGTGCAGCTGAAGCTTCGTACGC |
|  | BG26-OR | AAATTTTGACCCATGACGGCCAAACCAATCAAACCGAAATCAGCAGACATCACTATAGGGAGACCGGCAG |
|  | BG26-OCR | CAGTGAAACCGTGGTCAG |
| *SWI6* | BG27-OFII | GTCTTCATCTGGACCGTTTGGATAAGATCATTCACCAAATCTTAGAACTACTGTAAAGCGGCGGA |
|  | BG27-OR | GGGATCTCATTGTGAGGTCCTAAGTATCGTACCACTTCTTCCAACGCCATCACTATAGGGAGACCGGCAG |
|  | BG27-OCR | CAGTCTCCGAGTCTCTAGTG |
| *NKP2* | BG30-OF | TCAAGCAGCAAGTTATATAAAGTTGTTTCTCACAGCTTCTTGCACTCCCTTTACAGCAGCGCAGCTTAGCCAGCTGAAGCTTCGTACGC |
|  | BG30-OR | GTGAGTAGCGAATCCGAAACATAGTTATGCAGCAGCTGTTCAGAGTTCATCACTATAGGGAGACCGGCAG |
|  | BG30-OCR | CTCGTGTACGACTGTAGTTG |
| *HOM2* | BG32-OF | ACTACAATATATGCATATCAGTTAGAATTCTGACATCTGAACGTCTTCAGGACGTTTAGGCACTACTGCTCAGCTGAAGCTTCGTACGC |
|  | BG32-OR | CCAACGGAACCAGTAGCACCCAAAACACCAGCAATTTTCTTTCCAGCCATCACTATAGGGAGACCGGCAG |
|  | BG32-OCR | GAGGCACCAAGAACTTTCAG |
| *ALD6* | BG34-OF | GGATTCAAGACAAGCAACCTTGTTAGTCAGCTCAAACAGCGATTTAACGGTCTAAGCCGACAGGAGTCTCCAGCTGAAGCTTCGTACGC |
|  | BG34-OR | GGAAGTGTGATCTTGACTGGTTCAGCAGTGTCAAAGTGTAGCTTAGTCATCACTATAGGGAGACCGGCAG |
|  | BG34-OCR | GGTTGGTTGCTCGTATGTC |
| *SLA1* | BG37-OF | TAGAGAACATGCCTGCGTAGATTAAACCATGTAATTGATAGAGGTAGAATAGATGTAGACCACCGTGTCCCAGCTGAAGCTTCGTACGC |
|  | BG37-OR | TGCGGCTCATAGGCATAGACGGCCCTATAGATGCCCAGAAACACAGTCATCACTATAGGGAGACCGGCAG |
|  | BG37-OCR | TGGCCAGTTCTTCTGGTG |
| *SEC28* | BG38-OF | ATTCAATTGGAACCGTAATGTTGTATAAAATCTTTTGTCTTGCTGTATAGAGCACGTCTTCAATGCGAGCCAGCTGAAGCTTCGTACGC |
|  | BG38-OR | TGCACGAAGTTCCCCGTGTAGTAATTCTGCTTGATATTAAAGTAATCCATCACTATAGGGAGACCGGCAG |
|  | BG38-OCR | TTGCCAAGCTTAGAGGTAGG |
| *SNT309* | BG39-OF | GAGATAATTATACTTGTATATATAATTTGGGGCAGATAAGACTCCTGATAATTAGGCGACCACGAGCTTCCAGCTGAAGCTTCGTACGC |
|  | BG39-OR | TTGTAACCATCTGGTATTTTCCCTTTATCAACAAAGCTAAGGCCGTCCATCACTATAGGGAGACCGGCAG |
|  | BG39-OCR | TCGGGATGAACAGGCTCTC |
| *REG1* | BG40-OF | GTTCATCAGCTCTATCAAGATATAATATTTGCTCATCTTTATCTCTCTTAGGTGCTACCAACCAACTAGACAGCTGAAGCTTCGTACGC |
|  | BG40-OR | TTTTCAATATCTTTCTTACCGGCGAAGTAATTTGCTAGATTTGTTGACATCACTATAGGGAGACCGGCAG |
|  | BG40-OCR | CTTCGTCGTTGTCGTTATCG |
| *HTL1* | BG41-OF | CTGACAGTTTCAAGATGTCCATGACGCTTTGGTGCCCCTTACTAAGTTTGCTTGCGAGAGAACGAGATTACAGCTGAAGCTTCGTACGC |
|  | BG41-OR | TTGTAAGCTCTTTCAGGATTCATTGAGCTGATTGTGTTGTTCTGTGACATCACTATAGGGAGACCGGCAG |
| *POL32* | BG42-OF | GCCTCAATATAGAACACTTCATTAATGGAAGTACCTTAATAAGGTTATACATGACCGGAGCTTCCAGCATCAGCTGAAGCTTCGTACGC |
|  | BG42-OR | ACCTCAGTGAAGAGCTTCTCATTGATAAAATATGACGCCTTTTGATCCATCACTATAGGGAGACCGGCAG |
|  | BG42-OCR | GCCGATCTTCAAGTGGTG |
| *DHH1* | BG43-OF | CTCATCATTGGATTCAGTTTTGTTTCATCCACATTCTTTTAAGACCACAACACTGTGGACGATACGGTCTCAGCTGAAGCTTCGTACGC |
|  | BG43-OR | TCCGTATTACTGTTATTATTAGTGTTGAAGTTATTATTGATGGAACCCATCACTATAGGGAGACCGGCAG |
|  | BG43-OCR | CTGTCTGTGGTCTGGTATCC |
| *VRP1* | BG44-OF | TTGACAGAGGATGGAATCACAATGTTAGCCTTCAAATCCTACTATCCTGTACATAACTGGTTTCAAGGGCCAGCTGAAGCTTCGTACGC |
|  | BG44-OR | CCTAATGCTGGTGGAGGCGGTGGAGGAGGAGGAGCTGGAGCACCTGCCATCACTATAGGGAGACCGGCAG |
|  | BG44-OCR | TGCCGGTTTAGGAGCAC |
| *VPS15* | BG46-OFII | TGAAAAACAGATATATCTTAAGTAAGATACTTAAGCAGACAAGTTGAATTATTGTGAGGCACCGCCGAATCAGCTGAAGCTTCGTACGC |
|  | BG46-OR | ATGGCTATGGAAGGTGATGCTTGGACCACTAGTGATAATTGTGCCCCCATCACTATAGGGAGACCGGCAG |
|  | BG46-OCR | TTTCGCCGTTAGGGTCC |
| *ANP1* | BG51-OF | CGCCCGCCTTGTCTCATCGTGAAAAAATTGCGTAAGATGAAATCATGAAAATCACTGTAGTCACGTAGCTCAGCTGAAGCTTCGTACGC |
|  | BG51-OR | ATACTTACTGTGGTAGGGTTGAACGAGAGTTTTCTGTTATTATACTTCATCACTATAGGGAGACCGGCAG |
|  | BG51-OCR | GGGCTTGAAGATTCCTTGG |

**Table S5:** DNA cassettes used to reverse engineer the mutated alleles *rpn4-1* and *rtg1-1* in CEN.PK113-7D. The primers used to amplify the DNA cassettes used for the allele swapping are indicated as well as the primers used for the confirmation of the correct allele replacement and the primers used to confirm the mutation insertion by a High Resolution Melting analysis (HRM). The sequence of the primers are indictaed in Table S6.

| **Target gene** | **Cassettes** | **Primers amplification cassettes** | **SHR sequence** | **Primers confirmation insertion** | **Primers HRM** |
| --- | --- | --- | --- | --- | --- |
| *RPN4* | KanMX marker | KanRPN-F/ KanMXB-R | B | KanMX-DCF/  RPN-R | RPNCON-F/ RPN-R |
|  | Mutated *RPN4* allele | RPNB-F/ RPNDown-R |  |  |  |
| *RTG1* | KanMX marker | KanRTG-F/ KanMXA-R | A | KanMX-DCF/ RTGCON-R | RTGCON-F/ RTGCON-R |
|  | Mutated *RTG1* allele | RTGA-F/RTG-R |  |  |  |

**Table S6:** Primers used for the deletion of *URA3* and for the reverse engineering of the mutated alleles of *RPN4* and *RTG1* present in the evolved strains.

| **Primer name** | **Sequence** |  | |
| --- | --- | --- | --- |
| URA3-KanMXF | TTCTTAACCCAACTGCACAGAACAAAAACCTGCAGGAAACGAAGATAAATCCAGCTGAAGCTTCGTACGC | | Deletion |
| URA3-KanMXR | AGCTCTAATTTGTGAGTTTAGTATACATGCATTTACTTATAATACAGTTTTCTTTAAACACGGCCGCATAG | | Deletion |
| KanRPN-F | GCTTTTTAGTTGAACTAATAGTAGATAGTACTAATAGCTAATTTGTATCTTAGTTACGCTAGGGATAACAGGGTAATATAGCAGCTGAAGCTTCGTACGC | | Amplification of the KanMX cassettes including SHR B |
| KanMXB-R | GTTGAACATTCTTAGGCTGGTCGAATCATTTAGACACGGGCATCGTCCTCTCGAAAGGTGCATAGGCCACTAGTGGATCT | |  |
| RPNB-F | CACCTTTCGAGAGGACGATGCCCGTGTCTAAATGATTCGACCAGCCTAAGAATGTTCAACAGTTACGCTAGGGATAACAGGGTAATATAGTTAGGCGGTTACCATAACCC | | Amplification of the rpn4-1 cassette including SHRB |
| RPNDown-R | CTGCCGGACATGATTGAAG | |  |
| RPNCON-F | TCTGAGGGCTATCAGAAGAC | | Confirmation of RPN4 allele replacement. |
| RPN-R | TTCTTGACGTTGTTCTAACGAC | |  |
| KanRTG-F | TAAAAAGAATGGAATTAACTTCAACCACAACACAGACAAAAAACACTAGATAGAGTTACGCTAGGGATAACAGGGTAATATAGCAGCTGAAGCTTCGTACGC | | Amplification of the KanMX cassettes including SHR A |
| KanMXA-R | GTGCCTATTGATGATCTGGCGGAATGTCTGCCGTGCCATAGCCATGCCTTCACATATAGTCATAGGCCACTAGTGGATCT | |  |
| RTGA-F | ACTATATGTGAAGGCATGGCTATGGCACGGCAGACATTCCGCCAGATCATCAATAGGCACAGTTACGCTAGGGATAACAGGGTAATATAGGTGCACCATCATTGCCTTC | | Amplification of the *rtg1-1* cassette including SHRA |
| RTG-R | GTTTGCCCTTGTCTTGTAGC | |  |
| RTGCON-F | AGCAAGGCCAAAGGTACAG | | Confirmation of RTG1 allele |
| RTGCON-R | TATGTACTCTACCGCCTGTG | | replacement. |

**References**

1. de Kok,S, Nijkamp,JF, Oud,B, Roque,FC, de,RD, Daran,JM, Pronk,JT, van Maris,AJ: **Laboratory evolution of new lactate transporter genes in a j*en1Delta* mutant of *Saccharomyces cerevisiae* and their identification as *ADY2* alleles by whole-genome resequencing and transcriptome analysis**. *FEMS Yeast Res* 2012, **12**:359-374.
